# Pharmacogenetic testing for adverse drug reaction prevention: systematic review of economic evaluations and the appraisal of quality matters for clinical practice and implementation

**DOI:** 10.1186/s12913-021-07025-8

**Published:** 2021-10-02

**Authors:** Saowalak Turongkaravee, Jiraphun Jittikoon, Onwipa Rochanathimoke, Kathleen Boyd, Olivia Wu, Usa Chaikledkaew

**Affiliations:** 1 Social, Economic and Administrative Pharmacy (SEAP) Graduate Program, Department of Pharmacy, Faculty of Pharmacy, Mahidol University, Bangkok, Thailand; 2 Department of Biochemistry, Faculty of Pharmacy, Mahidol University, Bangkok, Thailand; 3 Health Economics and Health Technology Assessment (HEHTA), Institute of Health and Wellbeing, University of Glasgow, Glasgow, UK; 4 Social and Administrative Pharmacy Division, Department of Pharmacy, Faculty of Pharmacy, Mahidol University, 447 Sri-Ayuthaya Rd, Payathai, Ratchathewi, Bangkok, 10400 Thailand; 5 Mahidol University Health Technology Assessment (MUHTA) Graduate Program, Mahidol University, Bangkok, Thailand

**Keywords:** Pharmacogenomics, Adverse drug reactions, Economic-evaluation, Systematic review, Personalized medicine

## Abstract

**Background:**

Genetic testing has potential roles in identifying whether an individual would have risk of adverse drug reactions (ADRs) from a particular medicine. Robust cost-effectiveness results on genetic testing would be useful for clinical practice and policy decision-making on allocating resources effectively. This study aimed to update a systematic review on economic evaluations of pharmacogenetic testing to prevent ADRs and critically appraise the quality of reporting and sources of evidence for model input parameters.

**Methods:**

We searched studies through Medline via PubMed, Scopus and CRD’s NHS Economic Evaluation up to October 2019. Studies investigating polymorphism-based pharmacogenetic testing, which guided drug therapies to prevent ADRs, using economic evaluation methods were included. Two reviewers independently performed data extraction and assessed the quality of reporting using the Consolidated Health Economic Evaluation Reporting Standards (CHEERS) guidelines and the quality of data sources using the hierarchy of evidence developed by Cooper et al.

**Results:**

Fifty-nine economic evaluations of pharmacogenetic testing to avoid drug-induced ADRs were found between 2002 and 2018. Cost-utility and cost-effectiveness analyses were the most common methods of economic evaluation of pharmacogenetic testing. Most studies complied with the CHEERS checklist, except for single study-based economic evaluations which did not report uncertainty analysis (78%). There was a lack of high-quality evidence not only for estimating the clinical effectiveness of pharmacogenetic testing, but also baseline clinical data. About 14% of the studies obtained clinical effectiveness data of testing from a meta-analysis of case-control studies with direct comparison, which was not listed in the hierarchy of evidence used.

**Conclusions:**

Our review suggested that future single study-based economic evaluations of pharmacogenetic testing should report uncertainty analysis, as this could significantly affect the robustness of economic evaluation results. A specific ranking system for the quality of evidence is needed for the economic evaluation of pharmacogenetic testing of ADRs. Differences in parameters, methods and outcomes across studies, as well as population-level and system-level differences, may lead to the difficulty of comparing cost-effectiveness results across countries.

**Supplementary Information:**

The online version contains supplementary material available at 10.1186/s12913-021-07025-8.

## Background

In the United States (US), adverse drug reactions **(**ADRs**)** are the fourth to the sixth leading cause of death, with approximately more than 100,000 deaths per year [1]. Besides this, there is a similar trend in Europe, with approximately 5% of all hospitalizations and 197,000 deaths annually reported [2, 3]. The most severe life**-**threatening ADRs are the Stevens**-**Johnson syndrome **(**SJS**)** and toxic epidermal necrolysis **(**TEN**).** The majority of cases are caused by reactions to certain drugs, e.g., allopurinol, sulfa-drugs or carbamazepine**.** Moreover, ADRs could result in substantial economic burden. The annual economic impact of severe and fatal ADRs leading to mortality and morbidity was found to be exceptionally high, totaling nearly $177 billion in the US and €79 billion in Europe [2, 4].

Nowadays, there are several methods for investigating people who are at risk of ADRs according to clinical features, such as renal or liver function, age, dosage of administration, as well as identification of any drug interaction. Genetic factors can also be the cause of ADRs, which accounted for approximately 10–20% [5]. Genetic information obtained from polymorphism-based pharmacogenomics or pharmacogenetics is highly crucial to better identifying responders and non-responders to medications, as well as people who are at risk of ADRs or drug inefficacy prior to prescription [6]. Moreover, there has been an increasing number of genetic associations to develop clinically useful tests through international guidelines, including the Clinical Pharmacogenetics Implementation Consortium (CPIC), the Royal Dutch Association for the Advancement of Pharmacy - Pharmacogenetics Working Group (DPWG) and the Canadian Pharmacogenomics Network for Drug Safety (CPNDS).

The CPIC has developed guidelines on evidence-based pharmacogenetic testing for 54 drugs-gene pairs, which supported and guided the translation of clinically relevant aspect. In addition to this, pharmacogenetics-based therapeutic recommendations for 94 and 8 drugs-gene pairs were published by DPWG and the CPNDS, respectively [7]. It is important to note that the application of pharmacogenetic information before prescribing the corresponding medication is beneficial to avoid serious ADRs or to guide genotype-specific dosing, thereby enhancing the effective use of drug treatment. Therefore, national drug agencies have approved drug labels containing pharmacogenetic information. As of August 1, 2020, 335, 134, 105 and 52 drug labels were approved by the United States Food and Drug Administration (US FDA), the European Medicines Agency (EMA), the US Health Care Service Corporation (HCSC) and the Pharmaceuticals and Medical Devices Agency (PMDA) of Japan, respectively [6, 7].

Currently, the most important criteria for its implementation in clinical practice is not only clinical evidence of pharmacogenetic testing, but also its value for money, which can be proved by an economic evaluation being a vital tool used to inform resource allocation in the decision-making process, especially in developed countries [8, 9]. Therefore, the quality of methodological rigor in cost-effectiveness studies is required to increase the reliability of such studies. Until now, there were two systematic reviews specifically focusing on economic evaluation of pharmacogenetic testing to prevent ADRs [10, 11]. The first review published in 2008, [10] was aimed at determining the cost of thiopurine methyltransferase (*TPMT*) genotyping per averted case of neutropenia. It did not, however, evaluate the quality of included studies. Later in 2016, another review, [11] which included all studies up to 2015, assessed the quality of studies in terms of their reporting and evidence of clinical effectiveness of testing but did not include other parameters. Notably, it has been suggested that the sources of evidence for clinical effectiveness, baseline clinical value, resource utilization, cost and utility data, all of which can influence and contribute to biased estimates of economic evaluation results, should be taken into account [12].

Therefore, this review aimed to update a systematic review and critically appraise the quality of existing economic evaluations of pharmacogenetic testing to prevent ADRs, in terms of reporting and sources of evidence used for all significant model inputs, such as clinical effectiveness, baseline clinical data, resource use, cost and utilities. Due to methodological differences across studies, as well as population-level and system-level differences, our findings could assist in identifying the potential model parameters that could influence the cost-effectiveness results and their transferability across geographic regions. They could also be valuable in a future and robust cost-effectiveness analysis of pharmacogenetic testing to prevent ADRs, which might help policy-makers make better decisions on allocating resources effectively and implement such testing into clinical practice.

## Methods

A systematic review protocol was initially registered with PROSPERO, an international prospective registry of systematic reviews **(**identification number CRD42019142060) and available from: http**://**www**.**crd**.**york**.**ac**.**uk**/**PROSPERO**/**display_record**.**php?ID**=**CRD42019142060. The present systematic review was conducted in accordance with the guidelines of the Preferred Reporting Items for Systematic Reviews and Meta**-**Analysis **(**PRISMA**)** [13].

### Identification of studies

We conducted a systematic search in Medline (via PubMed), Scopus and the Centre for Reviews and Dissemination (CRD)‘s National Health Service Economic Evaluation Database (NHS EED) to identify relevant studies up to October 2019. The search terms were constructed based on the PICOS domains (patient, intervention, comparison, outcome and study type). The search terms were comprised of the domains on the intervention (pharmacogenetic testing and ADRs) and study type (economic evaluation). There were no restrictions in the domains of patients, comparators and outcomes. The search terms were explicitly used for each search engine and search strategies for each database, as stated in the **online appendices (Electronic Supplementary Material)** **Table A1**. The reference lists of the retrieved studies were also explored to identify further studies. The search was updated every six months.

### Selection of studies

Two reviewers (ST and OR) independently selected studies by screening titles and abstracts of all articles based on the eligibility criteria. Full texts of articles identified in the initial screening were retrieved. The studies were included if they met all of the following criteria. First, studies were included if they investigated pharmacogenetic testing of human genetic variations, which guided drug therapies to prevent ADRs. Second, the study type was an economic evaluation, e.g., cost-effectiveness analysis (CEA), cost-utility analysis (CUA), cost-benefit analysis (CBA) or cost-minimization analysis (CMA). Studies were excluded if the drug and pharmacogenetic testing were not on the list of the available clinical practice guidelines (e.g., CPIC, DPWG, CPNDS), or if the prescribing information for labelling was not approved by the US FDA as of August 1, 2020. Any disagreements were resolved through discussion.

### Data extraction

Data were extracted independently by two authors (ST and OR) using a data extraction form, which included the study characteristics, author, year of publication, setting, target populations, intervention, comparator, marker frequency, methods, perspective, time horizon, discounting, uncertainty analysis, and outcome measures, in terms of incremental cost, incremental cost per quality adjusted life year (QALY) or life year (LY) gained or cost per adverse reaction/event avoided**.** We also gathered the parameters, which may affect the cost-effectiveness results according to the uncertainty analysis results of individual studies.

### Quality assessment of economic evaluation reporting

The quality of economic evaluation reporting was appraised using the Consolidated Health Economic Evaluation Reporting Standards (CHEERS) checklist with 24 items [14]. Two independent authors (ST and OR) assessed the quality of reporting and any disagreements were resolved through discussion. A percentage of agreement and disagreement by checklist item was calculated. We evaluated the quality of reporting along with the CHEERS checklist by rating scores as follows: the study met all standards (score = 1), the study met some standards (score = 0.5), the study did not meet the standards (score = 0), or the study was not applicable (N/A). For instance, in the checklist item indicating whether the study reported time horizon and described its appropriateness, a score of 1, 0.5 and 0 will be given if the authors met all standards (i.e., they reported both the time horizon and reason why it was appropriate), if they met some standards (i.e., they reported either the time horizon or description of its appropriateness), and if they did not meet the standards (i.e., failed to report both).

### Quality assessment of evidence used

The quality of evidence for input parameters used in economic evaluations, such as clinical effect sizes, baseline clinical data, resource use, costs and utilities (for cost-utility analyses) was assessed using the hierarchy of data sources developed by Cooper et al. [12]. Each item was evaluated and given a rank ranging from 1 to 6, and 9 was applied to a source which was not clear. For example, for parameters related to clinical effect sizes, rank 1+ or 1 was given if the data were obtained from a meta-analysis of randomized controlled trials (RCTs) or single RCT with direct comparison measuring final outcomes, respectively. Other rates included: rank 2 (a single RCT with direct comparison measuring surrogate outcomes), rank 3 (a single placebo RCTs measuring surrogate outcomes), rank 4 (case control or cohort studies), rank 5 (case report or case series) and rank 6 (expert opinion). Two authors (ST and OR) independently assessed and ranked data sources of the input parameters based on the hierarchy of data sources published by Cooper et al. [12]. Any disagreements were resolved through discussion and a percentage of agreement and disagreement by input parameter was calculated.

### Transferability assessment of economic evaluation studies

We applied the transferability method developed by Welte et al. [15] to identify potential transferability factors across countries, which can be categorized into three groups: (1) methodology (i.e., perspective, time horizon, cost categories, and discount rate) (2) healthcare system (i.e., practice variation and technology availability), and (3) population characteristics (i.e., disease incidence/prevalence, life expectancy, acceptance and compliance). Based on our review, economic evaluation studies of pharmacogenetic testing conducted across countries were selected as a case-study to assess whether different transferability factors could directly affect the difference in costs and outcomes of the economic evaluation results.

## Results

### Search results

**Fig. 1:**
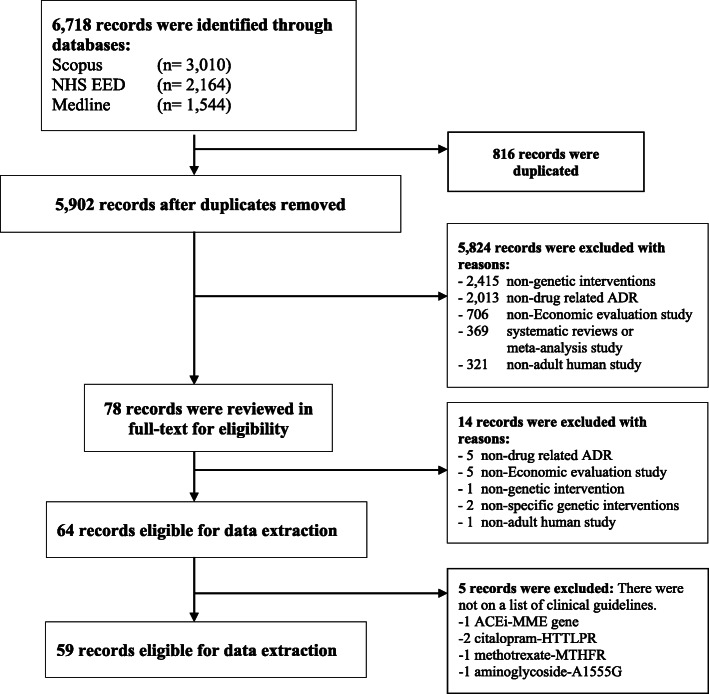
PRISMA flow of study selection process

A total of 6718 studies were searched from Medline (1544 studies), Scopus (3010 studies) and Cochrane Database of Systematic Reviews (CDSR) (2164 studies). After excluding 816 duplicates, 5902 studies were screened for titles and abstracts. From these, 5824 studies were excluded for several reasons. The most common reasons were “non-genetic interventions” and “non-drug-related ADRs”, as described in Fig. 1. A total of 64 studies met the inclusion criteria. Nevertheless, five studies were excluded since they were not included in the list of available clinical practice guidelines. Finally, 59 studies were eligible for data extraction.

### Characteristics of the included studies

The general characteristics of all included studies are presented in **online appendices (Electronic Supplementary Material)** **Table A2**. All studies were published between 2002 and 2018. CUA was the most frequent type of economic evaluation (41 studies) (70%), followed by CEA (10 studies) (17%), CBA (5 studies) (8%), and then CMA (3 studies) (5%). Table 1 demonstrates the number of studies categorized by therapeutic area-gene and ADRs, as well as by region. Majority of the studies were conducted in European and American countries (43 studies) (73%), while studies related to HLA-B*58:01-allopurinol and HLA-B*15:02-carbamazepine were mostly found in Asian countries. Most studies investigated the therapeutic area of cardiovascular diseases (24 studies) [16–39], followed by gout (8 studies) [40–47], human immunodeficiency virus (HIV) infection (8 studies) [48–55], autoimmune diseases (8 studies) [56–63], and epilepsy/neuropathic pain (6 studies) [64–69], cancer (3 studies) [70–72], major depressive disorder [73], and hormone replacement therapy [74].

**Table 1:** Number of studies classified by therapeutic area-gene and ADRs and by region

Therapeutic area-gene and ADRs				Clinical guideline	FDA approved labelling	Number of studies by region		
Drug	Gene	ADRs	Severity of ADRs			Asians	Europeans/ USA	Total
**Cardiovascular disease (24)**								
Warfarin	*CYP2C9 and VKORC1*	bleeding	NS	CPIC, DPWG, CPNDS	√	2	12	**14**
Clopidogrel	*CYP2C19*	major cardiac/adverse CV events	NS	CPIC,DPWG	√	1	8	**9**
Statins	Pharmacogenetics test	myopathy	NS	CPIC, DPWG	√	–	1	**1**
**Gout (8)**								
Allopurinol	HLA-B*58:01	SJS/TEN, DRESS	S	CPIC	√	6	2	**8**
**HIV infection (8)**								
Abacavir	HLA-B*57:01	Hypersensitivity	S	CPIC, DPWG	√	1	6	**7**
Efavirenz	*CYP2B6*	CNS toxicity	NS	CPIC,DPWG	√	–	1	**1**
**Autoimmune disease (8)**								
Azathioprine	*TPMT*	severe bone marrow toxicity	S	CPIC, DPWG	√	1	7	**8**
**Epilepsy/neuropathic pain (6)**								
Carbamazepine	HLA-B*15:02	SJS/TEN	S	CPIC, CPNDS	√	5	–	**5**
Carbamazepine	HLA-A*31:01	SJS/TEN, Hypersensitivity	S	CPIC, CPNDS	√	–	1	**1**
**Cancer (3)**								
Irinotecan	*UGT1A1*	severe neutropenia	S	DPWG	√	–	2	**2**
Fluoropyrimidines	*DPYD*	severe hematologic, GI toxicity	S	CPIC, DPWG	√	–	1	**1**
**Major depressive disorder (1)**								
Nortriptyline	*CYP2D6*	anticholinergic symptoms	NS	CPIC, DPWG	√	–	1	**1**
**Hormone replacement therapy (1)**								
Estrogen combined in oral contraceptives	Factor V Leiden	venous thromboembolic disease	NS	DPWG	–	–	1	**1**

**CBA:** cost-benefit analysis**, CEA:** cost-effectiveness, **CMA:** cost-minimization analysis, **CUA:** cost-utility analysis, **CPIC:** the Clinical Pharmacogenetics Implementation Consortium, **CPNDS:** the Canadian Pharmacogenomics Network for Drug Safety, **DPWG:** the Royal Dutch Association for the Advancement of Pharmacy - Pharmacogenetics Working Group, **DRESS:** drug reaction with eosinophilia and symptomatic symptoms, **FDA:** the United States Food and Drug Administration, **NS**: Non-Severe ADRs, **S**: Severe ADRs, **SJS:** Stevens-Johnson syndrome, **TEN:** toxic epidermal necrolysis

The majority of pharmacogenetic testing to prevent ADRs were *CYP2C9* and *VKORC1* testing before prescription of warfarin (14 studies) [16–29], *CYP2C19* genotype screening for selection of antiplatelet therapy (i.e., clopidogrel) after percutaneous coronary intervention (PCI) for acute coronary syndrome (ACS) patients (9 studies) [30–38], and HLA-B*58:01 screening before prescribing allopurinol in patients with gout (8 studies) [40–47]. Moreover, the severity of ADRs related to gene-drug pairs was grouped into two major types: severe ADRs (life-threatening or fatal ADRs) and common ADRs. Pharmacogenetic testing and drugs associated with severe ADRs were HLA-B*58:01-allopurinol induced SJS/TEN/drug reaction with eosinophilia and systemic symptoms (DRESS), HLA-B*57:01-abacavir induced hypersensitivity reaction, HLA-B*15:02 and HLA-A*31:01- carbamazepine induced SJS/TEN/hypersensitivity, *TPMT-*azathioprine induced severe bone marrow toxicity, *UGT1A1-* irinotecan induced severe neutropenia and *DPYD-* fluoropyrimidines induced severe hematologic/GI toxicity. Meanwhile, the others were pharmacogenetic testing and drug-associated common ADRs. Most of the genetic information regarding gene-drug pairs was published by the CPIC guideline [75] and drug labels were approved by the US FDA, except for the study determining Factor V Leiden screening before receiving estrogen combined with oral contraceptives [76].

### Quality assessment of economic evaluation reporting using the CHEERS checklist

The quality of economic evaluation reporting using the CHEERS checklist [14] is summarized in **online appendices (Electronic Supplementary Material)** **Table A3****.** All included studies clearly described the study population, measurement of effectiveness based on single study or synthesis estimated, as well as approaches for estimating resource use and costs with the percentage of agreement between two independent authors ranging from 81 to 100%, indicating that the score rating for each item was reliable. In contrast, only 22% of the single (trial) study-based performed uncertainty analysis of the parameters and evaluated their effects, as detailed in Table 2. Notably, 18 studies (31%) adopted a health service or healthcare payer’s viewpoint in the analysis, while 10 studies (17%) and 9 studies (15%) presented societal and healthcare system’s perspectives, respectively. There were nine studies (15%) that did not mention the study’s perspective [27, 28, 32, 41, 49, 57, 59, 66, 74]. The time horizon used for cost and consequence evaluation ranging from six weeks to a lifetime was also reported in the above studies, while five studies (8%) did not state the time horizon [32, 48, 61, 66, 70]. Among the studies that specified a time horizon exceeding one year, there were seven studies (18%) that did not report the discount rate for costs and outcomes [32, 39, 48, 49, 61, 66, 70].

**Table 2:** Quality assessment results of economic evaluation reporting using the CHEERS checklist

Item (Item No)	Number of studies met the recommendations	Percentage (%)	%Agreement†
Target population and subgroups (4)	59/59	100	98
Study perspective (6)	50/59	85	81
Comparators (7)	58/59	98	98
Time horizon (8)	54/59	92	98
Discount rate for costs and outcomes (9) ^††^	32/39	82	86
Measurement of effectiveness (Single study-based estimates) (11a)^#^	50/50	100	95
Measurement of effectiveness (Synthesis-based estimates) (11b) ^##^	9/9	100	95
Estimating resources and costs (Single study-based EE) (13a)*	9/9	100	95
Estimating resources and costs (Model-based EE) (13b)**	50/50	100	95
Study parameters (18)	50/59	85	84
Incremental costs and outcomes (19)	57/59	97	95
Characterizing uncertainty (Single study-based EE) (20a)*	2/9	22	98
Characterizing uncertainty (Model-based EE) (20b)**	50/50	100	100
Source of funding (23)	47/59	80	95
Conflicts of interest (24)	46/59	78	97

EE: Economic evaluation

† Percent agreement between two independent raters

†† The denominator for the calculations is the studies that reported the discount rate for costs and/or outcomes were from a study period longer than one year, including 39 studies

# The denominator for the calculations is a single study-based estimate of clinical effectiveness data, including 50 studies

## The denominator for the calculations is synthesis-based estimates of clinical effectiveness data, including 9 studies

* The denominator for the calculations is a single study-based economic evaluation, including 9 studies

** The denominator for the calculations is a single model-based economic evaluation, including 50 studies

For clinical effectiveness data, 50 studies (85%) used a single, study-based estimate and nine studies (15%) used synthesis-based estimates. All studies clearly described the source of evidence. Most studies (50 studies; 85%) were conducted based on the decision-analytic model, while nine studies (15%) used a single study-based economic evaluation. Seven studies (78%) including retrospective, observational, or RCT-based economic evaluations studies did not mention uncertainty analysis [27, 32, 41, 48, 59, 61, 66] as they did not indicate confidence intervals denoting uncertainty measures. However, all model-based economic evaluations performed uncertainty analysis. More than half (26 of 50 model-based studies; 52%), performed both one-way sensitivity analysis and probabilistic sensitivity analysis (PSA), while some conducted the one-way sensitivity analysis only (6 studies; 12%) and PSA only (6 studies; 12%). There were 12 studies (20%) and 13 studies (22%) that did not describe any source of funding [21, 28, 40, 41, 48, 51, 54, 57, 59–62] and the potential for conflict of interest of study contributors [29, 37, 43, 45, 52, 54, 57, 59–61, 63, 71, 74], respectively.

### Quality assessment of evidence used

Only 16 studies (27%) obtained clinical effectiveness data of testing from high-quality evidence (a single RCT with direct comparison, rank 1), while about half (49%) retrieved evidence from case-control or cohort studies (rank 4). Nevertheless, 8 studies (14%) used clinical effectiveness data of testing from the meta-analysis of case-control study with direct comparison, which was not listed in the hierarchy of data sources by Cooper et al. [12]. Moreover, only four (7%) and one studies (2%) applied the baseline clinical data from a high-quality evidence [case series specifically performed for the study (rank 1) and the analysis of administrative databases including only patients in interested settings (rank 2)]; whereas most studies (85%) obtained clinical effectiveness data of testing from old case series, analysis of reliable administrative databases, or estimated from RCTs (rank 4). On the other hand, most studies sourced resource use and cost information from a high-quality evidence [prospective data analysis conducted for specific study (rank 1) or recently published cost estimation based on reliable databases (rank 2)], except for one study on HLA-B*57:01-abacavir, which used data from expert opinions (rank 6) [52]. For CUA studies, the utility data were mostly (93%) estimated from a direct utility from a previous study in patients with the disease of interest (rank 3). Only one study for *CYP2B6*-efavirenz did not define the data source (rank 4) [53]. However, there was no evidence obtained from indirect utility or expert opinion. Overall, the percentage of agreement between two independent authors ranging from 81 to 97%, suggesting that the ranking of data sources was reliable, see Table 3**.**

**Table 3:** Quality assessment results of evidence used based on the hierarchy of data sources by Cooper et al. [12]

Input parameters	Hierarchy of data sources								% Agreement*
	1	2	3	4	5	6	9	#	
Clinical effect sizes	16 (27%)	0	0	29 (49%)	0	2 (3%)	4 (7%)	8 (14%)	85
Baseline clinical data	4 (7%)	1 (2%)	0	51 (85%)	1 (2%)	1 (2%)	1 (2%)		81
Resource use	7 (12%)	51 (86%)	0	0	0	1 (2%)	0		92
Costs	6(10%)	53 (90%)	0	0	0	0	0		85
Utility	2(5%)	0	38 (93%)	1 (2%)	0	0	0		97

# Meta-analysis of case-control with direct comparison between comparator therapies and measuring final outcomes

*Percentage of agreement between two independent raters

### Cost-effectiveness results

In terms of pharmacogenetic testing and drugs associated with ADRs in particular disease areas, such as cardiovascular diseases, gout, HIV infection, autoimmune diseases, epilepsy/neuropathic pain, cancer, major depressive disorder, and hormone replacement therapy, the results of economic evaluation studies are summarized and presented in Table 4.

**Table 4:** Cost-effectiveness results of included studies

No	Author, Year of published	study setting	Target populations	Intervention vs comparator	Cost effectiveness threshold	Cost-effectiveness results (ICER/ICUR)
**Drug: warfarin, Biomarker: CYP2C9 and VKORC1** **ADRs: bleeding events**						
1	Kim,DJ et al., 2017 [16]	Korea	mechanical heart valve replacement (MHVR)	(a) warfarin (b) CYP2C19 and VKORC1 genotyping-guided dosing of warfarin	$50,000 per QALY gained	• ICER (b) vs (a): $13,562 per QALY gained
2	Verhoef et al., 2016 [17]	UK and Sweden	Atrial Fibrillation (AF)	(a) warfarin (b) CYP2C19 and VKORC1 genotyping-guided dosing of warfarin	UK £20,000 per QALY gained, Sweden500,000 SEK	• In UK: ICER (b) vs (a): £6702 per QALY gained • In Sweden: ICER (b) vs (a): 253,848 SEK per QALY gained
3	Mitropoulou et al., 2015 [18]	Croatia	ischemic stroke patients with AF	(a) warfarin (b) CYP2C19 and VKORC1 genotyping-guided dosing of warfarin	€40,000 to €50,000 per QALY gained	• ICER (b) vs (a): €31,225 per QALY gained
4	Chong, HYet al, 2014 [21]	Thailand	newly initiated warfarin therapy	(a) warfarin (b) CYP2C19 and VKORC1 genotyping-guided dosing of warfarin	160,000 THB or $5333 per QALY gained	• Healthcare system perspective: ICER (b) vs (a):1,477,042 THB ($49,234) per QALY gained • Societal perspective ICER (b) vs (a): 1,473,852 THB ($49,128) per QALY gained
5	You, et al., 2014 [19]	USA	AF	(a) warfarin (b) CYP2C19 and VKORC1 genotyping-guided treatment	$50,000 per QALY gained	• ICER (b) vs (a): $ 2843 per QALY gained
6	Pink et al., 2014 [20]	Sweden	Non-valvular AF	(a) warfarin (b) CYP2C19 and VKORC1 genotyping-guided treatment (c) dabigatran (d) rivaroxaban (e) apixaban	£20,000–30,000 per QALY gained	• ICER (b) vs (a): £ 13,226 per QALY gained • ICER (e) vs (b): £20,671 per QALY gained • (d) is dominated by (c) and (e), high cost and lower QALYs than (c) and (e) • (c) is dominated by (e), high cost and lower QALYs than (e)
7	You et al., 2012 [22]	USA	newly diagnosed AF	(a) warfarin (b) CYP2C19and VKORC1genotyping-guided treatment (c) dabigatran 110 mg twice daily (d) dabigatran 150 mg twice daily	$50,000 per QALY gained	• (a) is dominated by (b), high cost and lower QALYs than (b) • ICER (d) vs (b): $13,810 per QALY gained • (c) is dominated by (d), (d) lower cost and more effective than (c)
8	Meckley et al., 2010 [23]	USA	newly initiated warfarin therapy	(a) warfarin (b) CYP2C19 and VKORC1 genotyping-guided dosing of warfarin	$50,000 per QALY gained	• ICER (b) vs (a): $ 60,725 per QALY gained
9	Eckman et al., 2009 [26]	USA	Non-valvular AF	(a) warfarin (b) CYP2C19 and VKORC1 genotyping-guided dosing of warfarin	$50,000 per QALY gained	• ICER (b) vs (a): $171,750 per QALY gained
10	Patrick et al., 2009 [25]	USA	newly diagnosed AF	(a) warfarin (b) CYP2C19 and VKORC1 genotyping-guided dosing of warfarin	$50,000 per QALY gained	• ICER (b) vs (a): ICER< 50,000 per QALY gained if it increased the time spent in the target INR range during the first 3 months of treatment by 5 to 9 percentage points
11	You et al., 2009 [24]	USA	newly initiated warfarin therapy	(a) warfarin (b) CYP2C19 and VKORC1 genotyping-guided dosing of warfarin	$50,000 per QALY gained	• ICER (b) vs (a): $ 347,059 per QALY gained
12	McWilliam et al., 2008 [27]	USA	newly initiated warfarin therapy	(a) warfarin (b) CYP2C19 and VKORC1 genotyping-guided dosing of warfarin	N/A	• Low baseline bleeding: ICER (b) vs (a): $82,890 per bleeding averted • Medium baseline bleeding: ICER (b) vs (a): $13,589 per bleeding averted • High baseline bleeding: (b) is dominated by (a) lower cost than (a)
13	Schalekamp et al., 2006 [28]	The Netherlands	newly initiated warfarin therapy	(a) warfarin (b) CYP2C19 and VKORC1 genotyping-guided dosing of warfarin	€20,000	• ICER (b) vs (a): € 4233 per bleeding averted
14	You et al., 2004 [29]	USA	newly initiated warfarin therapy	(a) warfarin (b) CYP2C19 and VKORC1 genotyping-guided dosing of warfarin	N/A	• ICER (b) vs (a): $5778 per bleeding averted
**Drug: clopidogrel, Biomarker: CYP2C19 ADRs: major cardiac/adverse cardiovascular events**						
1	Wang Y et al., 2018 [30]	Hong Kong	ACS undergoing PCI	(a) clopidogrel (b) ticagrelor (c) CYP2C19 testing guided therapy Test: positive: ticagrelor; Test negative: clopidogrel	$42,423 per QALY gained	• ICER (c) vs (a): $2560 per QALY gained • (b) is dominated by (c), high cost and lower QALYs than (c)
2	Jiang, M et al., 2017 [31]	USA	ACS undergoing PCI	(a) clopidogrel (b) prasugrel or ticagrelor (c) CYP2C19 testing guided therapy Test: positive: prasugrel or ticagrelor; Test negative: clopidogrel	$50,000 per QALY gained	• (a) is dominated by (c), high cost and lower QALYs than (c) • (b) is dominated by (c), high cost and lower QALYs than (c)
3	Deiman BA et al., 2016 [32]	the Netherlands	ACS undergoing PCI	(a) clopidogrel (b) prasugrel (c) ticagrelor (d) CYP2C19 testing guided therapy Test: positive: prasugrel or ticagrelor; Test negative: clopidogrel	€65,000 per QALY gained	• ICER (d) vs (a): €81,500 per QALY gained • ICER (d) vs (b): €9111 per QALY gained • ICER (d) vs (c): €5972 per QALY gained
4	Kazi DS et al., 2014 [34]	USA	ACS undergoing PCI	(a) clopidogrel (b) prasugrel (c) ticagrelor (d) CYP2C19 testing guided therapy Test: positive: prasugrel or ticagrelor; Test negative: clopidogrel	$50,000 per QALY gained	• Genotyping with prasugrel vs (a): $35,800 per QALY gained • Genotyping with ticagrelor vs (a): $30,200 per QALY gained
5	Patel et al., 2014 [33]	USA	ACS undergoing PCI	(a) clopidogrel+asprin (b) prasugrel+aspirin (c) CYP2C19 testing guided therapy Test: positive: prasugrel+asprin; Test negative: clopidogrel+asprin	$50,000 per QALY gained	• ICER (c) vs (a): $4200 per QALY gained • (b) is dominated by (c), high cost and lower QALYs than (c)
6	LALA A et al., 2013 [36]	USA	ACS undergoing PCI	(a) clopidogrel (b) prasugrel (c) CYP2C19 testing guided therapy Test: positive: prasugrel; Test negative: clopidogrel	$50,000 per QALY gained	• (a) is dominated by (c), (c) lower cost and more effective than (a) • (b) is dominated by (c), (c) lower cost and more effective than (b)
7	Sorich et al., 2013 [35]	Australia	ACS undergoing PCI	(a) clopidogrel (b) ticagrelor (c) CYP2C19 testing guided therapy Test: positive: ticagrelor; Test negative: clopidogrel	AUS$ 50,000 per QALY gained	• ICER (c) vs (a): $AUS 6346 per QALY gained • ICER (b) vs (c): $AUS 22,821 per QALY gained
8	Panattoni L et al., 2012 [38]	New Zealand	ACS (included the four largest ethnic groups)	(a) clopidogrel (b) prasugrel (c) CYP2C19 testing guided therapy Test: positive: prasugrel; Test negative: clopidogrel	$NZ50000 per QALY gained	• (b) is dominated by (a), high cost and lower QALYs than (a) • ICER (c) vs (a): $NZ 24,617 per QALY gained • (b) is dominated by (c), (c) lower cost and more effective than (b)
9	Reese E S et al., 2012 [37]	USA	ACS, recent MI or stroke undergoing PCI	(a) clopidogrel (b) prasugrel (c) CYP2C19 testing guided therapy Test: positive prasugrel; Test negative clopidogrel	N/A	• (a) is dominated by (c), (c) lower cost and more effective than (a) • (b) is dominated by (c), (c) lower cost and more effective than (b)
**Drug: statin, Biomarker: pharmacogenetics test ADR: myopathy, rhabdomyolysis**						
1	Mitchel et al., 2017 [39]	Canada	cardiovascular patients	(a) statin (b) genotyping-guided treatment with statin (only patients experiencing musculoskeletal pain are being tested)	CAN $6150 per QALY gained	• (a) is dominated by (b), high cost and lower QALYs than (b) • (b) would be cost-effective as long as the test costs less than CAN$906
**Drug: allopurinol, Biomarker: HLAB-5801** **ADR: SJS/TEN, DRESS**						
1	Cheng H et al., 2018 [41]	China (Han population)	Hyperuricemia and gout	(a) allopurinol 100 mg and 600 mg per day (b) febuxostat 40mgand 80 mg per day (c) HLA-B5801 testing prior to treatment Test positive: febuxostat Test negative: allopurinol	N/A	• In all 253 patients • (c) saved 1,384,040 yuan for allopurinol and febuxostat at the lowest dosages • (c) saved 2,807,770 yuan for allopurinol and febuxostat at the highest dosages
2	Chong et al., 2018 [40]	Malaysia	gout	(a) allopurinol starting dose 300 mg, target dose 600 mg per day (current practice) (b) probenecid target dose 2 g per day (c) HLA-B5801 testing prior to treatment Test positive: probenecid Test negative: allopurinol	MYR 39,000 or $8695 per QALY gained	• (b) is dominated by (a), high cost and lower QALYs than (a) • (c) is dominated by (a), high cost and lower QALYs than (a)
3	Jutkowitz et al., 2017 [44]	USA	gout	(a) allopurinol-febuxostat sequential therapy: allopurinol:target dose 300 mg/day and febuxostat: 80 mg/day, (current practice) (b) HLA-B5801 testing prior to treatment Test positive: febuxostat Test negative:allopurinol	$109,000 per QALY gained	• ICER (b) vs (a): for -Asians $64,190, -African Americans $83,450, -Caucasians or Hispanics $183,720 per QALY gained
4	Ke CH et al., 2017 [43]	Taiwan	gout with chronic kidney disease	(a) benzbromarone 100 mg/day (current practice) (b) allopurinol target dose 100 mg/ day (c) febuxostat 80 mg/day (d) HLA-B5801 testing prior to treatment Test positive: febuxostat or benzbromarone Test negative: allopurinol	NT $800,000 or US $25,600 per QALY gainedin 2015	• (b) is dominated by (a), high cost and lower QALYs than (a) current practice) • (c) is dominated by (d), high cost and lower QALYs than (d) current practice) • ICER (d) vs (a): NT$ 234,610 per QALY gained in the base-case and NT$ 230,925 per QALY gained in patients with chronic kidney disease
5	Plumpton CO et al., 2017 [42]	UK	gout	(a) allopurinol target dose 300 mg per day add prophylactic treatment with colchicine (b) HLA-B5801 testing prior to treatment Test positive: febuxostat: target dose 80 mg per day Test negative: allopurinol	£30,000 per QALY gained	• ICER (d) vs (a): £44,954 in the base case and £38,478 per QALY in patients with chronic renal insufficiency
6	Dong D et al., 2015 [46]	Singapore	gout	(a) allopurinol starting dose 300 mg, target dose 600 mg per day (b) allopurinol +safety program (SP) (c) HLA-B5801 testing prior to treatment + SP (d) HLA-B5801 testing prior to treatment + SP Test positive: probenecid target dose 2 g per day Test negative: allopurinol (e) HLA-B5801 testing prior to treatment Test positive: probenecid target dose 2 g per day Test negative: allopurinol (f) no allopurinol (treatment of acute flares only)	$50,000 per QALY gained	• -ICER (b) vs (a): $79,140 per QALY • (c) is dominated by (b), high cost and lower QALYs than (b) current practice) • ICER (d) vs (b): $85,630 per QALY • (e) is dominated by (d), high cost and lower QALYs than (d) current practice) • (f) is dominated by (d), high cost and lower QALYs than (d) current practice)
7	Park DJ et al., 2015 [45]	Korea	gout with chronic renal insufficiency	(a) allopurinol starting dose 100 mg, target dose 300 mg per day (b) HLA-B5801 testing prior to treatment Test positive: febuxostat starting dose 40 mg, target dose 80 mg) per day Test negative: allopurinol	N/A	• Costs of (a) $1193 and (b) $1055 (b) is less costly and more effective than (a)
8	Saokaew et al., 2014 [47]	Thailand	gout	(a) allopurinol starting dose 300 mg per day (b) HLA-B5801 testing prior to treatment Test positive: probenecid target dose 1 mg target to 2 g per day Test negative: allopurinol	160,000 THB per QALY gained	• ICER (b) vs (a): 156,937 THB per QALY
Drug: abacavir, Biomarker: HLA B*57:01 **ADR: hypersensitivity**						
1	Kubaeva et al., 2018 [48]	Russia	HIV	(a) abacavir regimen (b) HLA-B* 5701 testing prior to treatment Test positive: alternative regimens without abacavir Test negative: abacavir regimen	N/A	• (b) vs (a): was cost-saving 54,1646 rubles
2	Kapoor R et al., 2015 [49]	3 ethnic groups in Singapore	HIV	(a) abacavir-based ART (b) tenofovir-based ART (c) HLA-B:5701 testing prior to treatment Test positive: tenofovir-based ART Test negative: abacavir-based ART	$50,000 per QALY gained	• **Early stage:** ICER (c) vs (a): Chinese $415,845 per QALY gained, Malay $318,029 per QALY gained, Indian $208,231 per QALY gained • **Late stage:** ICER (c) vs (a): Chinese $926,938 per QALY gained, Malay $624,297 per QALY gained, Indian $284,598 per QALY gained • **HIV who are contraindicated to tenofovir** ICER (b) vs (a): all ethnicity was not cost-effective, except for Indian patients with early-stage ICER (c) vs (a): $44,649 per QALY gained
3	Nieves Calatrava et al., 2010 [51]	Spain	HIV	(a) abacavir regimen (b) HLA-B:5701 testing prior to treatment Test positive: alternative HAART regimen without ABC Test negative: abacavir regimen	N/A	• cost per HSR avoided of €630
4	Kauf TL et al., 2010 [52]	USA	HIV	(a) short-term: abacavir+ lamivudine+efavirenz (b) long-term: tenofovir+emtricitabine+efavirenz (c) HLA-B:5701 testing prior to treatment Test positive: tenofovir+emtricitabine+efavirenz Test negative: abacavir+lamivudine+efavirenz;	$50,000 per QALY gained	• Short-term: (c) is dominated by (a), high cost and lower QALYs than (a) • Long-term: (b) is dominated by (c), high cost and lower QALYs than (c)
5	Wolf et al., 2010 [50]	Germany	HIV	(a) combination of abacavir+ lamivudine (b) HLA-B:5701 testing prior to treatment Test positive combination of tenofovir, emtricitabine Test negative combination of abacavir+ lamivudine	N/A	• (b) vs (a): (b) cost-saving €44 and 127 per screened patient from healthcare payer and societal perspective
6	Schackman BR et al., 2008 [53]	USA	HIV	(a) abacavir-based regimen (b) tenofovir-based regimen (c) HLA-B:5701 testing prior to treatment Test positive: tenofovir or AZT-based regimen Test negative: abacavir-based regimen	$50,000 per QALY gained	• ICER (c) vs (a): $36,700 per QALY gained • (b) is dominated by (a),high cost and lower QALYs than (a)
7	Hughes DA et al., 2004 [54]	UK	HIV	(a) Trizivir (AZT/3TC/ABC) (b) HLA-B:5701 testing prior to treatment Test positive: HAART regimen without abacavir Test negative: Trizivir (AZT/3TC/ABC) (HAART: highly active antiretroviral therapy)	N/A	• (a) is dominated by (b), high cost and lower benefit than (b), except for Trizivir is substituted with ritonavir+indinavir+combivir • ICER (b) vs (a): € 22,811 per hypersensitivity reaction avoided
**Drug: efavirenz, Biomarker: CYP2B6, ADRs: sub- or supratherapeutically dosed**						
1	Schackman et al., 2015 [55]	USA	HIV	(a) efavirenz 600 mg + tenofovir+ emtricitabine (b) CYP 2B6 testing prior to treatment Test positive: decrease dose to 200,400 mg Test negative: efavirenz 600 mg (c) Universal low dose efavirenz 400 mg + tenofovir+ emtricitabine	$ < 100,000 per QALY gained	• (a) is dominated by (b), high cost and lower benefit than (b)
**Drug: azathioprine, Biomarker: TPMT ADR: neutropenia or severe neutropenia**						
1	Thompson AJ et al., 2014 [56]	UK	autoimmune diseases	(a) azathioprine therapy (b) TPMT testing prior to treatment Test positive: alternative treatment Test negative: azathioprine	£20,000 per QALY gained	• (b) is dominated by (a) lower cost and lower QALYs than (a)
2	Priest VLet al, 2006 [58]	New Zealand	inflammatory bowel disease (IBD)	(a) azathioprine (b) TPMT testing prior to treatment (Phenotype and genotype)	N/A	• Phenotype and genotype testing dominated by (a), high cost and lower QALYs than (a) There are cost-savings (vs no testing) of $NZ120,000 and 71 neutropenias avoided;-034QALYs and $NZ11,000 and 40 neutropenias avoided, −058QALYs)
**Drug: azathioprine, Biomarker: TPMT ADR: leukopenia**						
3	Hagaman JTet al, 2010 [57]	USA	Idiopathic Pulmonary Fibrosis (IPF)	(a) conservative therapy which no specific therapy (b) azathioprine+N-acetylcysteine and steroids (no testing) (c) TPMT testing prior to treatment Test positive: conservative therapy Test negative: azathioprine+N-acetylcysteine and steroid	$50,000 per QALY gained	• ICER (b) vs (a): $49,245 per QALY *ICER (c) vs (b): $29,663 per QALY gained
4	Dubinsky MC et al., 2005 [60]	USA	Crohn’s disease	(a) TPMT testing prior to treatment Test positive: metrotrexate Test negative: azathioprine (b) metabolite monitoring (MM) prior to treatment with azathioprin (c) TPMT testing +MM prior to treatment with azathioprine (d) community care (CC)	N/A	• (d) is dominated by (a), (b),and (c), (d) less costs and faster time to response or sustained response
5	Winter J et al., 2004 [61]	Scotland	IBD	(a) azathioprine (b) TPMT testing prior to treatment	N/A	• for a 30 year old: ICER (b) vs (a): £347 per life-year saved and • for a 60 year old: ICER (b) vs (a): £817 per life-year saved
**Drug: azathioprine, Biomarker: TPMT ADR: all adverse events**						
6	Sayani FA et al., 2005 [59]	Canada	Crohn’s disease and IBD	(a) azathioprine (b) TPMT testing prior to treatment	N/A	• The direct health care costs for (a) $30,011 per patient and (b) $34,887 per patient
**Drug: azathioprine, Biomarker: TPMT ADR: severe bone marrow toxicity**						
7	Oh KT et al., 2004 [62]	Korea	rheumatoid arthritis and systemic lupus erythematosus	(a) azathioprine (b) TPMT testing prior to treatment	N/A	• (a) is dominated by (b), (b) lower cost and more effective than (a)
**Drug: azathioprine, Biomarker: TPMT ADR: hematological cytopenias**						
8	Marra CA et al., 2002 [63]	Canada	rheumatoid arthritis and systemic lupus erythematosus	(a) azathioprine (b) TPMT testing prior to treatment	N/A	• (a) cost $677 Cdn per patient, (b) cost $663 Cdn per patient
Drug: carbamazepine, Biomarker: HLAA*15:02, ADRs: SJS, TENs, hypersensitivity						
1	Chong et al., 2017 [64]	Malaysia	Epilepsy	(a) carbamazepine (current practice) (b) sodium valproate (VPA) (c) HLA-B*15:02 testing prior to treatment Test positive: VPA Test negative: carbamazepine	MYR 37,000 ($ 8982) per QALY gained	• (b) is dominated by (a), high cost and lower QALYs than (a) current practice) • (c) is dominated by (a), high cost and lower QALYs than (a) current practice)
2	Chen et al., 2016 [65]	Hong Kong	Epilepsy	(a) current situation, using antiepileptic drug (pre-policy period) (b) current situation, using antiepileptic drug (post-policy period) (c) HLA-B*15:02 testing prior to treatment (the ideal situation) (ideal situation) Test positive: alternative anti-epileptic drug Test negative: carbamazepine or phenytoin (d) HLA-B*15:02 testing prior to either carbamazepine or phenytoin (extended situation)	$50,000 per QALY gained	• ICER (b) vs (a): $85,697 per QALY gained • ICER (c) vs (a): $11,090 per QALY gained • ICER (d) vs (a): $197,158 per QALY gained
3	Rattanavipapong W et al., 2013 [67]	Thailand	Epilepsy or neuropathic pain	(a) carbamazepine (no HLA-B*15:02 testing) (b) HLA-B*15:02 testing prior to treatment Test positive: for epilepsy: valproate, for neuropathic pain: gabapentin; Test negative: carbamazepine (c) all prescribed alternative (no HLA-B*15:02 testing) (epilepsy: valproate, neuropathic pain: gabapentin)	120,000 THB per QALY gained	• ICER (b) vs (a): 222,000 THB per QALY for epilepsy, 130,000 THB per QALY for neuropathic pain • ICER (c) vs (a): 32,522,000 per QALY for epilepsy, (epilepsy), 35,877,000 per QALY for neuropathic pain
4	Tiamkao S et al., 2013 [66]	Thailand	Epilepsy or neuropathic pain and neurological diseases	(a) carbamazepine (b) HLA-B*15:02 testing prior to treatment Test positive: non-specified Test negative: carbamazepine	N/A	• (b) vs (a): (b) was cost-saving 98,54,994 THB per 100 cases of carbamazepine users
5	Dong D et al., 2012 [68]	Singapore	Epilepsy	(a) carbamazepine or phenytoin (b) HLA-B*15:02 testing prior to treatment Test positive: valproate; Test negative carbamazepine or phenytoin (c) valproate (no screening)	$50,000 per QALY gained	ICER (b) vs (a): Chinese patients $37,030 per QALY gained, Malay $7930 per QALY gained and Indians $136,630 per QALY gained • (c) is dominated by (b), high cost and lower QALYs than (b)
Drug: carbamazepine, Biomarker: HLAA*31:01, ADRs: SJS, TENs, hypersensitivity						
1	Plumpton et al., 2015 [69]	UK	Epilepsy	(a) carbamazepine (b) HLA-B*31:01 testing prior to treatment Test positive: lamotrigine; Test negative: carbamazepine	£20,000 per QALY gained	• ICER (b) vs (a): £ 12,808 per QALY gained
Drug: Fluoropyrimidines, Biomarker: DPYD*2A genotype–guided dosing ADR: toxicity iehematologic, GI						
1	Deenen MJ et al., 2016 [70]	The Netherland	cancer	(a) fluoropyrimidines-based therapy (b) DPYD*2A testing prior to treatment Test positive: alternative regimen Test negative: Fluoropyrimidines-based	N/A	• (b) vs (a): (b) cost-savings of €45 ($61) per patient
**Drug: irinotecan, Biomarker: UGT1A1 ADR: severe neutropenia**						
1	Pichereau S et al., 2010 [71]	France	metastatic colorectal cancer	(a) FOLFIRI regimen (5-fluorouracil, leucovorin and irinotecan) (b) UGT1A1 testing prior to treatment with FOLFIRI Test positive: FOLFOX regimen (oxaliplatine + 5-FU + folinic acid) Test negative: FOLFIRI	N/A	• ICER (b) vs (a): to avoid one febrile neutropenia per 1000 patients treated was € 9428 to € 10,901
2	Gold HT et al., 2009 [72]	USA	metastatic colorectal cancer	(a) FOLFIRI regimen (5-fluorouracil, leucovorin and irinotecan) (b) UGT1A1 testing prior to treatment with FOLFIRI Test positive: reduce dose to intermediate dose Test negative: FOLFIRI	$100,000 per QALY gained	• ICER (b) vs (a): was cost-effective if the treatment efficacy of irinotecan in homozygotes after dose reduction had to be ≥984% of full-dose efficacy for genetic testing to remain preferred
**Drug: nortriptyline, Biomarker: CYP2D6 ADRs: sub- or supratherapeutic dose**						
1	Bern EJ et al., 2016 [73]	The Netherland	major depressive disorder	(a) nortriptyline (b) CYP2D6 genotyping-guided dosing of nortriptyline	€50,000 per QALY gained	• ICER (b) vs (a): €1,333,000 per QALY gained
**Drug: oral contraceptive, Biomarker: factor V Leiden ADR: Thromboembolism**						
1	Smith KJ, et al., 2008 [74]	USA	women who receiving oral contraceptives (OCPs)	(a) usual care (b) genotyping (c) genotyping with OCP counseling (d) genotyping with OCP counseling and AC for high-risk events (e) genotyping with OCP counseling and AC for long-term	$20,000 per QALY gained	• ICER (d) vs (c): $ 147 per QALY gained • ICER (e) vs (d): $ 639,500 per QALY • (a) is dominated by (d), high cost and lower QALYs than (d) • (b) is dominated by (d), high cost and lower QALYs than (d))

**AC:** anticoagulation, **ACS:** Acute Coronary Syndrome, **CBA:** Cost-benefit analysis, **CEA:** Cost-effectiveness analysis, **CMA:** Cost-minimization analysis, **CUA:** Cost-utility analysis, **EE:** Economic evaluation, **ICER:** Incremental Cost-Effectiveness Ratio

**OCP:** oral contraceptive pill, **PCI:** Percutaneous Coronary Intervention, **PSA:** Probabilistic Sensitivity Analysis

**QALY:** Quality adjusted life-year, **N/A:** Not Applicable, **WTP:** Willingness to pay

#### Cardiovascular diseases

##### *CYP2C9 and VKORC1* and warfarin-induced risk of bleeding

There were 14 economic evaluation studies of *CYP2C9* and *VKORC1* testing before prescription of warfarin to prevent risks of bleeding. Over the prior from 2004 to 2017, these studies were conducted in Korea (1 study) [16], UK and Sweden (1 study) [17], Croatia (1 study) [18], Thailand (1 study) [21], Sweden (1 study) [20], Netherlands [28] and US (8 studies) [19, 22–27, 29]. Ten studies were CUA with model-based economic evaluation [16–19, 21–26] and another study was CUA with a trial-based [20] economic evaluation to estimate resource use associated with the interventions. Two studies were CEA with model-based [28, 29] and one study was CEA based on a retrospective study [27]. Seven studies were explicitly conducted in patients with atrial fibrillation and one study investigated mechanical heart valve replacement (MHVR). The rest of the studies were used in newly initiated warfarin therapy. Nine studies showed that *CYP2C9* and *VKORC1* testing to prevent the risk of bleeding would be a cost-effective intervention [16–20, 22, 27–29] (i.e., less costly and more effective than treatment without genotyping). One study suggested that testing would be cost-effective if it increased the time spent in the target international normalized ratio (INR) range during the first three months of treatment by 5 to 9% [25]. Nevertheless, four studies from Thailand [21] and US [23, 24, 26] suggested that those testings would not be cost-effective due to the effectiveness of testing in reducing out-of-range INR values.

##### *CYP2C19* and clopidogrel-induced major adverse cardiovascular events (MACE)

There were nine studies which conducted an economic evaluation of *CYP2C19* testing before prescription of clopidogrel to avoid cardiovascular events in patients with ACS undergoing PCI. They were performed in Hong Kong (1 study) [30], Netherlands (1 study) [32], Australia (1 study) [35], New Zealand (1 study) [38] and US (5 study) [31, 33, 34, 36, 37]. They were conducted from 2012 to 2018. Seven studies were CUA with model-based approach [30, 31, 33–36, 38] and one study was CUA with trial-based [32] Others were CEA with model-based [37] economic evaluation. All studies showed that *CYP2C19* testing would be a potentially cost-effective treatment strategy for avoiding MACE. Nevertheless, some studies considered ticagrelor and/or prasugrel as alternative drugs with a higher cost than clopidogrel for those who tested positive.

##### Pharmacogenetic testing and statin-induced myopathy

One study developed testing that identified statin-induced myopathy in cardiovascular patients by using CUA with model-based economic evaluation in 2017. The results demonstrated that genotyping would be a cost-effective intervention, with a testing cost of CAN$ 906 that was less than the cost of no testing in Canada [39].

#### Gout

##### HLA-B*58:01 and allopurinol-induced SJS/TEN and DRESS

There were eight economic evaluation studies of HLA-B*58:01 screening before prescribing allopurinol in gout patients to prevent SJS/TEN and DRESS) that were performed in China (1 study) [41], Malaysia (1 study) [40], US (1 study) [44], Taiwan (1 study) [43], UK (1 study) [42], Singapore (1 study) [46], Korea (1 study) [45], and Thailand (1 study) [47] from 2014 to 2018. Six studies used CUA with model-based economic evaluation [40, 42–44, 46, 47]), one performed CMA with trial-based [41] and one conducted CBA with model-based approach [45]. All studies were conducted in patients with gout, and two studies were explicitly conducted in gout patients with Chronic Kidney Disease (CKD) [43, 45]. Most of the studies considered allopurinol-induced SJS/TEN, but only one study considered both SJS/TEN and DRESS. Five studies applied febuxostat as an alternative drug in the model [41–45] to patients who tested positive with HLA-B*58:01. However, probenecid has been used in Malaysia, Thailand and Singapore [40, 46, 47] as febuxostat is not regularly used in the usual clinical practice.

Three studies showed that HLA-B*58:01 genotyping would be a cost-effective in China, Taiwan and Thailand [41, 43, 47] and cost-saving intervention in the Korean study [45]. Nevertheless, three studies from Malaysia [40], UK [42] and Singapore [46] suggested that HLA–B*58:01 genotyping would not be cost-effective as the cost of the pharmacogenomics testing and alternative drugs, such as febuxostat were too high, and the efficacy of alternative drugs was less than that of allopurinol (e.g., probenecid). Moreover, the study in US [44] showed that genotyping would be cost-effective for Asians and African Americans. However, it would not be cost-effective for Caucasians or Hispanics. Therefore, the incremental cost-effectiveness ratios (ICERs) might vary substantially across racial or ethnic groups, following by their HLA-B*5801 frequency.

#### HIV infection

##### HLA-B*57:01 and abacavir-induced hypersensitivity reaction

There were seven economic evaluation studies of HLA-B*57:01 screening before prescribing abacavir for HIV positive patients to prevent hypersensitivity reactions (HSR) that were conducted in Russia (1 study) [48], Singapore (1 study) [49], Spain (1 study) [51], Germany (1 study) [50], UK (1 study) [54], and US (2 studies) [52, 53] from 2004 until 2018. Most studies were conducted in Europe and US [48, 50–54], and only one study investigated the Asian population [49]. Results showed that allele frequencies in Europe and US were much higher (ranged from 3.7–7.3%) than those of the Asian population (1.1% in Han Chinese, 1.8% in Malays), except for Indians (3.6%). Three studies were CUA with model-based [49, 52, 53], two studies were CEA with model-based [51, 54] and one was CBA with model-based economic evaluation [50]. The rest was CMA based on a retrospective study [48]. Four studies in Russia, Germany, UK and US demonstrated that HLA-B*57:01 testing would be cost-effective [53, 54] and cost-saving [48, 50] to prevent HSR due to abacavir as compared with no testing, while the remaining studies showed that it was not cost-effective [51, 52]. In addition, the study in Singapore [48] suggested that genotyping was not cost-effective for Han Chinese and Malays ethnicity but cost-effective in Indian patients. This was because the frequency of the HLA-B*5701 allele and positive predictive value (PPV) in Indians were higher than in Han Chinese and Malays.

##### *CYP2B6* and efavirenz-induced CNS toxicity

The study in US demonstrated that the *CYP2B6* genotyping before prescribing efavirenz to prevent central nervous system (CNS) toxicity in HIV patients was cost-saving as compared with no testing due to a lower lifetime cost and a gain in QALYs [55].

#### Autoimmune diseases

##### *TPMT* and azathioprine-induced severe bone marrow toxicity

Azathioprine-induced severe bone marrow toxicity was associated with *TPMT* in patients with autoimmune diseases, inflammatory bowel disease, idiopathic pulmonary fibrosis (IPF), Crohn’s disease, rheumatoid arthritis or systemic lupus erythematous. Eight studies were carried out in UK (1 study) [56], New Zealand (1 study) [58], Scotland (1 study) [61], Korea (1 study) [62], Canada (2 studies) [59, 63], and US (2 studies) [57, 60] from 2002 to 2014. These studies employed CUA with model-based (2 studies), [57, 58] CUA with trial-based (1 study) [56], CEA with model-based (2 studies), [60, 62] CEA with trial-based (1 study) [61], CBA with model-based (1 study) [63], and CMA with a randomized prospective study (1 study) [59]. Five studies showed that testing would be a cost-effective [57, 60–62] and cost-saving intervention [63] to prevent severe ADRs regarding azathioprine as compared with no testing. Nevertheless, two studies from UK [56] and New Zeland [58] suggested that genotyping would not be cost-effective due to higher costs and lower QALYs than azathioprine therapy without testing. In Canada, it was discovered that genetic testing was not cost-saving [59].

#### Epilepsy/neuropathic pain

##### HLA-B*15:02 and carbamazepine-induced SJS/TEN

Five economic evaluation studies of HLA-B*15:02 genotyping to prevent the risk of SJS/TEN in patients prescribed carbamazepine (CBZ) were carried out in Malaysia [64], Hong Kong [65], Thailand (2 studies) [66, 67] and Singapore [68] from 2012 to 2017. Four studies applied CUA with model-based economic evaluation [64, 65, 67, 68], while the other study used CBA with retrospective study [66]. All studies focused on the patients diagnosed with epilepsy. Only the study from Thailand [67] included both patients with epilepsy and neuropathic pain. Moreover, the study from Singapore [68] was performed separately for the major ethnic groups, which were Han Chinese, Malays and Indians. Three studies used valproate [64, 67, 68], while the rest of the studies used any anti-epileptic drug as an alternative for those patients who tested positive with HLA-B*15:02 [65, 66].

The findings from three studies showed that a testing would be cost-effective [65, 68] and cost-saving to prevent SJS/TEN in CBZ, as compared with no testing [66]. However, a study in Malaysia indicated that testing would not be cost-effective as a result of ethnicity and an effective alternative drug for those who tested positive [64]. The study in Thailand showed that HLA-B*15:02 screening would be cost-effective in CBZ-treated patients with neuropathic pain but not for epilepsy because the cost of alternative drugs for epilepsy was approximately two times higher than the cost for neuropathic pain [67].

##### HLA-A*31:01 and carbamazepine-induced SJS/TEN and hypersensitivity

Notably, CBZ has been associated with HLA-A*31:01 and it can lead to severe ADRs, such as SJS/TEN and hypersensitivity. A study in UK was performed using CUA with model-based economic evaluation in 2015. The results showed that testing would be cost-effective as the efficacy (e.g., remission rate) of anti-epileptic drugs was the main driver of cost-effectiveness results [69]. In addition, this study used lamotrigine as an alternative drug for patients who tested positive rather than valproate, which might be different from other clinical settings.

#### Cancer

##### *UGT1A1* and irinotecan-induced severe neutropenia

One CUA with model-based study from France [71] and one CEA with model-based study from the US [72] were performed to evaluate the cost-effectiveness of *UGT1A1* screening before prescribing irinotecan to prevent severe neutropenia in metastatic colorectal cancer. The results demonstrated that genotyping would be a cost-effective intervention.

##### *DPYD* and fluoropyrimidines-induced severe hematologic and GI toxicity

One study was conducted in the Netherlands [70] using CBA with model-based economic evaluation in 2016. The results demonstrated that *DPYD* testing before prescription of fluoropyrimidines would be cost-saving, as compared with no testing, in preventing severe hematologic and GI toxicity due to fluoropyrimidine.

#### Major depressive disorder

##### *CYP2D6* and nortriptyline-induced anticholinergic symptoms

The CUA with model-based economic evaluation in the Netherland study showed that *CYP2D6* screening for adjusting dose before starting nortriptyline compared to no screening would not be cost-effective since *CYP2D6* was not potentially related to the reduction of ADRs and to the increased efficacy of nortriptyline in a major depressive disorder [73].

#### Hormone replacement therapy

##### Factor V Leiden and estrogen combined in oral contraceptives-induced thromboembolism

The CUA with model-based study in US was conducted to evaluate the cost-effectiveness of Factor V Leiden testing before a prescription of estrogen-containing oral contraceptives to avoid thromboembolism. The study compared testing before prescribing the drug, testing with oral contraceptive pill (OCP) counselling, testing with OCP counselling and anticoagulation (AC) with the usual care without testing. The results demonstrated that testing with OCP counselling and prophylactic AC during high-risk periods in female relatives of FVL carriers was cost-effective [74].

### Uncertainty analysis results

Based on the results of uncertainty analysis from the included studies, parameters which could influence the cost-effectiveness results are summarized in terms of therapeutic areas and gene-drug pairs in Table 5. These parameters were classified into three types: (1) epidemiological and disease progression parameters, e.g., probability of ADRs related to drug treatment, allele frequency, PPV or negative predictive value (NPV), and mortality rate of ADRs, (2) clinical effectiveness data, e.g., the efficacy of genetic testing and drugs treatment, and (3) resource use and cost parameters, e.g., costs of genetic testing, alternative drugs and hospitalization**.**

**Table 5:** Number of studies reporting parameters which could influence the cost-effectiveness results

Therapeutic area	Epidemiological and disease progression parameters				Clinical effectiveness data		Resource use and cost parameters		
	probability of ADRs	allele frequency	PPV/NPV	mortality rate of ADRs	efficacy of genetic testing	efficacy of drugs treatment	cost of testing	costs of alternative drugs	cost of hospitalization
**Cardiovascular disease (*n*** **= 24)**									
*CYP2C19 -*Clopidogrel	[30, 33, 34]			[31]		[35]		[36]	[38]
*CYP2C9 and VKORC1* -Warfarin	[17, 21, 25]				[16, 22, 26, 28, 29]		[23, 24]	[19]	
pharmacogenetic testing-Statin						[39]			
**Gout (*n*** **= 8)**									
HLA-B*58:01 -Allopurinol	[43, 44, 46]	[45]				[40]	[42]	[44]	[40, 47]
**HIV infection (*n*** **= 8)**									
HLA-B*57:01 -Abacavir		[50]		[49]			[51, 52]	[53, 54]	
*CYP2B6 -*Efavirenz							[55]		
**Autoimmune disease (n = 8)**									
*TPMT -* Azathioprine		[57]	[62]		[63]		[56, 58]	[60]	
**Epilepsy/neuropathic pain (*n*** **= 6)**									
HLA-B*15:02 or HLA-A*31:01 -Carbamazepine			[67, 68]			[64, 69]		[65, 67]	
**Cancer (*n*** **= 3)**									
*UGT1A1 -*Irinotecan					[71]	[72]			
*DPYD* -Fluoropyrimidine	[70]								
**Major depressive disorder (*n*** **= 1)**									
*CYP2D6* -Nortriptyline					[73]	[73]			
**Hormone replacement therapy (n = 1)**									
Factor V Leiden-Estrogen combined in oral contraceptives									

**ADRs:** Adverse Drug Reactions, **PPV:** Positive Predictive Value, **NPV:** Negative Predictive Value

Our review indicated that cost-effectiveness results were mostly sensitive to the probability of drug induced-ADRs, the effectiveness of pharmacogenetic testing to prevent ADRs, the cost of testing, and the cost of alternative drugs in patients who tested positive. For instance, in cardiovascular diseases, the probability of MACE due to clopidogrel and the efficacy of *CYP2C9* and *VKORC1* testing to avoid bleeding complications of warfarin mostly affected the ICER results in clopidogrel and warfarin users, respectively. Furthermore, for HIV infection, the cost of testing had an impact on the ICER results in both abacavir and efavirenz. However, there was no reported uncertainty analysis from a one-way sensitivity analysis among these studies [18, 20, 32, 37, 41, 48, 59, 61, 66, 74].

### The transferability of economic evaluation results

Based on Welte et al’s method [15] that assesses the transferability of economic evaluation results across countries, three transferability factors were determined from the economic evaluations for HLAB*5801-allopurinol in gout patients [40–47] as a case study. First, methodological characteristics, e.g., perspective, time horizon, cost categories, and discount rate used, varied across CUA studies. Among six CUA studies, a healthcare payer perspective was the most common, followed by a societal perspective. Nevertheless, lifetime horizon was mostly applied. Cost categories and discount rates used were different. Although three main direct medication costs, e.g., the cost of HLAB*5801 testing, cost of treating ADRs and cost of gout maintenance treatment, were mostly included, the cost of flare management of an acute flare or death was considered in some studies. Based on a societal perspective, direct non-medical cost, e.g., transportation cost and additional food cost for patients and their relatives, and indirect costs, e.g., productivity loss due to illness, were incorporated. Costs and outcomes were discounted at a rate of 3% or 3.5%.

Secondly, the healthcare system characteristics in a particular practice varied among countries. It was reported that China, Taiwan, Korea, UK and US applied febuxostat as an alternative drug in the model based on the recommendations of the American College of Rheumatology that allopurinol and febuxostat were first-line agents for the management of gout [77, 78]. However, febuxostat is not regularly used as an alternative drug in the general clinical practice in Malaysia, Thailand and Singapore. Although the same alternative drug was used, the dosage differed across studies. For instance, allopurinol was used starting at either 100 to 600 mg/day or 100 to 300 mg/day in patients with CKD, febuxostat was used at 40 to 80 mg/day, and probenecid was used at 2 g/day.

Lastly, in terms of population characteristics, disease prevalence was one of the substantial variation factors that could not be transferred from one country to another. The HLA-B*5801 allele frequency and PPV for SJS/TEN were the key drivers influencing cost-effectiveness results. Interestingly, the study in US revealed that genotyping would be cost-effective for Asians and African Americans but not for Caucasians or Hispanics because the HLA-B*5801 frequency was varied substantially across racial or ethnic groups which had an impact on the ICERs [44]. Indeed, the HLA-B*5801 allele frequency ranged from 11.9–18.5% in Asian studies [40, 41, 43, 45–47] and was higher than in US and Europe [42, 44], which ranged from 0.7–3.8%.

Furthermore, the PPV in the Asians was higher than in American and European populations. This implied that Asians who carried HLA-B*5801 allele would have more chances to develop SJS/TEN as compared with Americans and Europeans. In summary, regarding the differences in three potential transferability factors across countries, the cost-effectiveness results would be useful for a context specific setting as they may not be directly transferred from one country to another.

## Discussion

Our study provided the most updated systematic review on economic evaluation studies of pharmacogenetic testing for prevention of ADRs (59 studies) as compared with two previously published systematic reviews in 2008 (7 studies) and 2016 (47 studies). The majority of included studies were conducted in cardiovascular diseases and mostly found in Europe and US; whereas, only one-third of them were performed in Asian countries. Given the fact that the frequency of each genotyping was different across countries, the cost-effectiveness of pharmacogenetic testing would depend on the ethnicity of patients who were receiving the tests. For instance, HLA-B*15:02 allele is more frequent among Asians than Caucasians, while HLA-A*31:01 is rarer in Asians, but more frequent in Caucasians. Therefore, the regular screening before starting carbamazepine therapy for HLA-B*15:02 in Asians is more useful than HLA-A*31:01 in the context of clinical implementation and future economic evaluation study. Alongside this, pharmacogenetic testing can prevent drug-induced severe ADRs on clinical outcomes and reduce economic burden, which are considered significant impacts involving the interest of policy-makers and healthcare professionals [2, 4]. Compared with previous published reviews, we included additional economic evaluation studies of other pharmacogenetic testing, such as *CYP2D6*-nortriptyline, *CYP2B6*-efavirenz, *DPYD*- fluoropyrimidines and *UGT1A1*- irinotecan, and statins.

Our review suggested that CUA and CEA were the most common methods for performing the economic evaluation of pharmacogenetic testing. This is consistent with the recommendation by Col NF et al. [79] and Payne K et al. [80], denoting that economic evaluation methods, i.e., CUA or CEA could capture all relevant costs and benefits of pharmacogenetics testing [79, 80]. In addition, our review on cost-effectiveness results of the pharmacogenetic testing for prevention of ADRs showed differences in the parameters, methods and outcomes among included studies. Consequently, this raised concerns on the transferability of the cost-effectiveness analysis results from one country to another, which has been increasingly recognized due to healthcare resource constraints [15].

Notably, our systematic review shed light on the critical appraisal of all included studies to evaluate the quality in terms of reporting and the source of evidence used for important model input parameters, which had significant impact on cost-effectiveness results. Based on the quality appraisal on reporting economic evaluations according to the CHEERS checklist [14], most studies complied with the checklist, except for single study-based economic evaluations. The finding highlighted that 78% of the single study-based economic evaluation studies did not report uncertainty analysis results of the parameters affecting cost-effectiveness results. This may be due to the fact that these reports did not indicate confidence intervals which are necessary measures for performing uncertainty analysis. It should be noted that the advantages of the uncertainty analysis surrounding effects and costs are to provide a correct evaluation of the expected effects and costs, to consider whether existing evidence is sufficient, and to assess the possible consequences of an uncertain decision for decision makers [81]. Therefore, it is worth noting that future studies on the economic evaluation of pharmacogenetic testing with single study-based studies should include uncertainty analysis, since this could significantly lead to the robustness of economic evaluation results. Furthermore, our study revealed that there were studies that failed to report funding (20%) and authors’ disclosure of conflicts of interest (COI) (22%), possibly leading to biased results when making decisions by clinicians, patients and policy-makers, as the authors or funders might have influenced the research findings. Most studies with the omissions of funding sources (67%) and COI (85%) were published between 2002 to 2010, when the reporting of this information had not been mandatory by the journal standards.

In addition, our review highlighted two gaps of knowledge that should be considered for assessing the quality of data sources used for pharmacogenetic testing. First, data sources of clinical effectiveness in several therapeutic areas related to pharmacogenetic testing to prevent drug-induced serious ADRs were very limited, which was consistent with the previous review [11]. Nevertheless, we appraised broader data sources of evidence used not only clinical effectiveness data, but also baseline clinical values, costs as well as resources used and utility data. Our results revealed that there was lack of high-quality evidence, not only estimating the clinical effectiveness of pharmacogenetic testing, but also providing baseline clinical data, according to the hierarchy of evidence developed by Cooper et al. [12]. For example, only 16 studies (27%) obtained clinical effectiveness data of genetic testing from five major RCTs: the PREDICT-1 trial [82] for HLA-B*57:01-abacavir, the TARGET trial [83] for *TPMT-*azathioprine, ARIES trial [84] and the COUMAGEN trial [85] for *CYP2C9 and VKORC1*-warfarin, the PLATO trial [86, 87] for *CYP2C19*-clopidogrel. Yet, of all RCTs, only two RCTs supported pharmacogenetic testing to prevent severe ADRs induced by abacavir and azathioprine. Interestingly, we found that eight studies (14%) obtained clinical effectiveness of testing from the meta-analysis of case-control study with direct comparison, which is not listed in the hierarchy of data sources by Cooper et al. [12].

Second, there were very limited baseline clinical data on pharmacogenetic testing. Our review revealed that only five studies (9%) explicitly analyzed baseline clinical data from reliable databases, including patients from the study setting given that such specific database included patients who developed severe ADRs, which are rare events that might not be commonly available. It should be noted that the quality of sources, especially for clinical effectiveness and baseline clinical data, used to evaluate the economic evaluations of pharmacogenetic testing would be relatively different from that of pharmaceutical interventions. Consequently, this could shed light on a specific ranking system for quality of evidence which is needed for economic evaluation of pharmacogenetic testing to prevent ADRs.

Our study had several strengths. First, pharmacogenetic testing and drug-related ADRs were selected based on the list of currently available clinical guidelines and approved drug labels. Thus, only studies related to treatment options in clinical practice were included to ensure a significant benefit of pharmacogenetic testing and might be useful for clinical decision-making and policy implementation. Second, we added several pharmacogenetic testing from previous studies, such as *CYP2D6*-nortriptyline, *CYP2B6*-efavirenz, *DPYD*-fluoropyrimidines, *UGT1A1*- irinotecan, and pharmacogenetic testing for statins. Third, we appraised the quality of included studies for both the quality of reporting and data sources of evidence used which had a broader component than the previous studies. This review also described in detail the differences in parameters, methods and economic evaluation results of included studies. Furthermore, we demonstrated a case study to evaluate the transferability of the study results across countries according to potential transferability factors to inform that the economic evaluations of pharmacogenetic testing would be useful for a specific setting or might not been transferred to other clinical settings. The above hypotheses have been supported by the clearly established evidence demonstrating that race/ethnicity and geographic region were possible influencers on the prevalence of HLA-B*5801, in which the prevalence of HLA-B*5801 (< 1%) was found to be lower in Caucasians and Hispanics than that in African Americans (3.8%) and Asians (7.4%) [1, 14].

It is significant to address some limitations in our study. First, we evaluated the quality of data sources for model input parameters from the only existing published criteria for economic evaluation study developed by Cooper et al. and the quality of reporting using the CHEERS checklist guidelines. However, the ranking of data sources may not be specific to the economic evaluations of pharmacogenetic testing. To the best of our knowledge, there have been existing published guidelines of the International Society for Pharmacoeconomics and Outcomes Research relevant to this topic [88–92], in which we did not apply them to our study. It is recommended that those guidelines could be used as criteria in future studies. Second, some studies did not report uncertainty analysis results, which could affect cost-effectiveness results, therefore we could consider only the results of one-way sensitivity analysis obtained from included studies.

## Conclusions

This comprehensive review found fifty-nine economic evaluations of pharmacogenetic testing to avoid drug-induced severe ADRs, which mostly focused on therapeutic areas of cardiovascular diseases. CUA and CEA were commonly applied to perform the economic evaluation of pharmacogenetic testing to prevent drug-induced ADRs. Based on the quality appraisal on reporting economic evaluations according to the CHEERS checklist guidelines [14], most studies complied with the guidelines, except that uncertainty analysis of single study-based economic evaluations should be reported. The quality of evidence used in clinical effectiveness data and the baseline clinical data were considered to be low-quality according to the hierarchy of evidence proposed by Cooper et al. Therefore, the criteria for assessing the quality of evidence used for economic evaluation of pharmacogenetic testing of ADRs are needed to be further developed. Differences in parameters, methods and outcomes across studies as well as population-level and system-level differences may lead to the difficulty of comparing cost-effectiveness results across countries. Our findings might be useful for developing future and robust cost-effectiveness analyses of pharmacogenetic testing to inform policy-makers on how to allocate resources effectively and implement such testing into clinical practice.

## Supplementary Information

**Additional file 1 Table S1.** Search strategies A) Search strategies in MEDLINE via PubMed and CRD’s NHS Economic Evaluation Database (NHS EED) and B) Search strategies in Scopus. **Table S2.** General characteristics and results of the included studies. **Table S3.** The assessment of quality of reporting using CHEERS checklist [14].

## Data Availability

Not Applicable.

## Abbreviations

ADRs: Adverse drug reactions
CHEERS: Consolidated Health Economic Evaluation Reporting Standards
SJS: Stevens**-**Johnson syndrome
TEN: Toxic epidermal necrolysis
CPIC: Clinical Pharmacogenetics Implementation Consortium
DPWG: Royal Dutch Association for the Advancement of Pharmacy - Pharmacogenetics Working Group
CPNDS: Canadian Pharmacogenomics Network for Drug Safety
US FDA: United States Food and Drug Administration
EMA: European Medicines Agency
HCSC: US Health Care Service Corporation
PMDA: Pharmaceuticals and Medical Devices Agency
PRISMA: Preferred Reporting Items for Systematic Reviews and Meta**-**Analysis
CRD: Centre for Reviews and Dissemination
NHS EED: National Health Service Economic Evaluation Database
CEA: Cost-effectiveness analysis
CUA: Cost-utility analysis
CBA: Cost-benefit analysis
CMA: Cost-minimization analysis
QALY: Quality adjusted life year
LY: Life year
N/A: Not applicable
RCTs: Randomized controlled trials
CDSR: Cochrane Database of Systematic Reviews
HIV: Human immunodeficiency virus
DRESS: Drug reaction with eosinophilia and systemic symptoms
OCP: Oral contraceptive pill
PPV: Positive predictive value
ICER: Incremental cost-effectiveness ratio
