# Supplementary material for: Pharmacogenetic testing for adverse drug reaction prevention: systematic review of economic evaluations and the appraisal of quality matters for clinical practice and implementation
Source: BMC Health Serv Res. 2021 Oct 2;21:1042. doi: 10.1186/s12913-021-07025-8 (PMC8487501; doi:10.1186/s12913-021-07025-8)
Supplement: Supplementary file 1 — Additional file 1 Table S1. Search strategies A) Search strategies in MEDLINE via PubMed and CRD’s NHS Economic Evaluation Database (NHS EED) and B) Search strategies in Scopus. Table S2. General characteristics and results of the included studies. Table S3. The assessment of quality of reporting using CHEERS checklist [14]. [file 12913_2021_7025_MOESM1_ESM.pdf]

Pharmacogenetic Testing for Adverse Drug Reaction Prevention: Systematic Review of Economic Evaluations and the Appraisal of Quality Matters for Clinical Practice and Implementation

Saowalak Turongkaravee[1], Jiraphun Jittikoon[2], Onwipa Rochanathimoke[1], Kathleen Boyd[3], Olivia Wu[3], Usa Chaikledkaew[4,5]

1. Social, Economic and Administrative Pharmacy (SEAP) Graduate Program, Faculty of Pharmacy, Mahidol University, Bangkok, Thailand

2. Department of Biochemistry, Faculty of Pharmacy, Mahidol University, Bangkok, Thailand

3. Health Economics and Health Technology Assessment (HEHTA), Institute of Health and Wellbeing, University of Glasgow, Glasgow, UK

4. Social and Administrative Pharmacy Division, Department of Pharmacy, Faculty of Pharmacy, Mahidol University, Bangkok, Thailand

5. Mahidol University Health Technology Assessment (MUHTA) Graduate Program, Mahidol University, Bangkok, Thailand

Corresponding author:

Usa Chaikledkaew, PhD.

Social and Administrative Pharmacy Division, Department of Pharmacy, Faculty of Pharmacy, Mahidol University

447 Sri-Ayuthaya Rd, Payathai, Ratchathevee, Bangkok, 10400, THAILAND

Tel. (66+) 26448694, Fax. (66+) 26448694

Email: usa.chi@mahidol.ac.th

ORCID iD:

Saowalak Turongkaravee: 0000-0003-1897-3495

Usa Chaikledkaew : 0000-0001-9457-9823

Supplementary material

Table A1. Search strategies A) Search strategies in MEDLINE via PubMed and CRD's NHS Economic Evaluation Database (NHS EED) and B) Search strategies in Scopus

Table A2. General characteristics and results of the included studies

Table A3. The assessment of quality of reporting using CHEERS checklist [14]

Table A1. Search strategies A) Search strategies in MEDLINE via PubMed and CRD's NHS Economic Evaluation Database (NHS EED) and B) Search strategies in Scopus

A) MEDLINE via PubMed and CRD's NHS Economic Evaluation Database (NHS EED)

Domains	Search	Query
I (1)	1	**"Genetic Testing" [Mesh]**
	2	**"Pharmacogenomic Testing" [Mesh]**
	3	**pharmacogenetics [Mesh]**
	4	biomarkers [Mesh]
	5	Search pharmacogenetic*
	6	Search Pharmacogenomic*
	7	Search pharmacogen*
	8	Search genomic*
	9	Search genetic*
	10	Search genotype*
	11	Search "single nucleotide polymorphism*"
	12	Search SNP
	13	Search biomarker*
	14	Search polymorphism
	15	Search Screen*
	16	Search Test*
	17	**#1 OR #2 OR #3 OR #4**
	18	**#5 OR #6 OR #7 OR #8 OR #9 OR #10 OR #11 OR #12 OR #13 OR #14 OR #15 OR #16**
	19	**#17 OR #18**
I (1)	20	**"Adverse Drug Reaction" ("Drug-Related Side Effects and Adverse Reactions" [Mesh])**
	21	Search "Adverse Drug Reaction*"
	22	Search "Drug Reaction*"
	23	Search "adverse drug event*"
	24	Search "Drug event*"
	25	Search "adverse effect*"
	26	Search "side effect*"
	27	Search "undesirable effect*"
	28	Search tolerability
	29	Search harm*
	30	Search toxic*
	31	Search toxicity

	32	**#21 OR #22 OR #23 OR #24 OR #25 OR #26 OR #27 OR #28 OR #29 OR #30 OR #31**
	33	**#20 OR #32**

**S**	34	**economic evaluation ("Cost-Benefit Analysis"[Mesh])**
	35	**pharmacoeconomic ("Economics, Pharmaceutical"[Mesh])**
	36	Search "economic evaluat*"
	37	Search pharmacoeconomic*
	38	Search cost-benefit
	39	Search Cost-effective*
	40	Search cost-utility
	41	**#34 OR #35**
	42	**#36 OR #37 OR #38 OR #39 OR #40**
	43	**#41 OR #42**
	44	**#19 AND #33 AND #43**
		**Filters: Humans; English**

B) Search strategies in SCOPUS

Domains	Search	Query
**I (1)**	1	Search pharmacogenetic*
	2	Search Pharmacogenomic*
	3	Search pharmacogen*
	4	Search genomic*
	5	Search genetic*
	6	Search genotype*
	7	Search "single nucleotide polymorphism*"
	8	Search SNP
	9	Search biomarker*
	10	Search polymorphism
	11	Search Screen*
	12	Search Test*
	13	**#1 OR #2 OR #3 OR #4 #5 OR #6 OR #7 OR #8 OR #9 OR #10 OR #11 OR #12**

**I (2)**	14	Search "Adverse Drug Reaction*"
	15	Search "Drug Reaction*"
	16	Search "adverse drug event*"
	17	Search "Drug event*"
	18	Search "adverse effect*"
	19	Search "side effect*"
	20	Search "undesirable effect*"
	21	Search tolerability
	22	Search harm*
	23	Search toxic*

	24	Search toxicity
	25	**#14 OR #15 OR #16 OR #17 OR #18 OR #19 OR #20 OR #21 OR #22 OR #23 OR #24 OR #25 OR**
**S**	26	Search "economic evaluat*"
	27	Search pharmacoeconomic*
	28	Search cost-benefit
	29	Search Cost-effective*
	30	Search cost-utility
	31	**#26 OR #27 OR #28 OR #29 OR #30**
**I&S**	**32**	**#13 AND #25 AND #31**
		**Filters: Humans; English, not article , not review after 1998**

Table A2. General characteristics and results of the included studies

No.	Author , Year of published	study setting	Target populations	Intervention vs comparator	Marker prevalence (%)	Perspective used	Trial- or Model-based EE	Methods	Time horizon	Discounting	Type of uncertainty analysis	WTP threshold	ICER/ICUR	Main outcome (finding)
**Drug: clopidogrel,<br>Marker: CYP2C19,<br>ADRs: major cardiac/adverse cardiovascular events**														
1	Wang Y. et l.,2018	Hong Kong	ACS undergoing PCI	(a). clopidogrel<br>(b). ticagrelor<br>(c).CYP2C19 testing guided therapy<br>Test: positive : ticagrelor;<br>Test negative : clopidogrel	51.8%	Health care provider	Model-based EE	CUA with decision tree and markov model	Lifetime	3%	one-way sensitivity analyses and PSA	$42,423 per QALY	*ICER (c) vs. (a): $2,560 per QALY<br>*(b) is dominated by (c), high cost and lower QALYs than (c)	Genetic testing was cost-effective
2	Jiang, M. et l.,2017	USA	ACS undergoing PCI	(a). clopidogrel<br>(b). prasugrel or ticagrelor<br>(c). CYP2C19 testing guided therapy<br>Test: positive: prasugrel or ticagrelor;<br>Test negative: clopidogrel	1.LOF 27.8%,<br>2.GOF among non-LOF allele carriers 40.6%	healthcare provider	Model-based EE	CUA with decision tree and markov model	30 years	3%	one-way sensitivity analyses and PSA	$50,000 per QALY	*(a) is dominated by (c), high cost and lower QALYs than (c)<br>*(b) is dominated by (c), high cost and lower QALYs than (c)	Genetic testing was cost-effective
3	Deiman BA et al. ,2016	the Netherlands	ACS undergoing PCI	(a). clopidogrel<br>(b). prasugrel<br>(c). ticagrelor<br>(d). CYP2C19 testing guided therapy<br>Test: positive: prasugrel or ticagrelor;<br>Test negative: clopidogrel	N/A	No data	Trial-based EE	CUA with observational study	N/A	N/A	No data	€65,000 per QALY	*ICER (d) vs. (a): €81,500 per QALY<br>*ICER (d) vs. (b): €9,111 per QALY<br>*ICER (d) vs. (c): €5,972 per QALY	Genetic testing was cost-effective
4	Kazi D.S.et al. ,2014	USA	ACS undergoing PCI	(a). clopidogrel<br>(b). prasugrel<br>(c). ticagrelor<br>(d).CYP2C19 testing guided therapy<br>Test: positive: prasugrel or ticagrelor;<br>Test negative: clopidogrel	N/A	Societal	Model-based EE	CUA with markov model	Lifetime	3%	Scenario sensitivity analyses and PSA	$50,000 per QALY	*Genotyping with prasugrel vs. (a): $35,800 per QALY<br>*Genotyping with ticagrelor vs. (a): $30,200 per QALY	Genetic testing was cost-effective
5	Patel et al.,2014	USA	ACS undergoing PCI	(a). clopidogrel+asprin<br>(b). prasugrel+aspirin<br>(c). CYP2C19 testing guided therapy<br>Test: positive: prasugrel+asprin ;<br>Test negative: clopidogrel+asprin	30.54%	healthcare provider	Model-based EE	CUA with markov model	15 months	5%	one-way sensitivity analyses and PSA	$50,000 per QALY	*ICER (c) vs. (a): $4,200 per QALY<br>*(b) is dominated by (c), high cost and lower QALYs than (c)	Genetic testing was cost-effective

No.	Author , Year of published	study setting	Target populations	Intervention vs comparator	Marker prevalence (%)	Perspective used	Trial- or Model-based EE	Methods	Time horizon	Discounting	Type of uncertainty analysis	WTP threshold	ICER/ICUR	Main outcome (finding)
6	LALA A. et al., 2013	USA	ACS undergoing PCI	(a). clopidogrel<br>(b). prasugrel<br>(c). CYP2C19 testing guided therapy<br>Test: positive: prasugrel;<br>Test negative: clopidogrel	27%	Payer	Model-based EE	CUA with decision tree and markov model	15 months	3%	one-way sensitivity analyses and PSA	$50,000 per QALY	*(a) is dominated by (c) , (c) lower cost and more effective than (a)<br>*(b) is dominated by (c) , (c) lower cost and more effective than (b)	Genetic testing was cost-effective
7	Zorich et al., 2013	Australia	ACS undergoing PCI	(a). clopidogrel<br>(b). ticagrelor<br>(c). CYP2C19 testing guided therapy<br>Test: positive : ticagrelor;<br>Test negative : clopidogrel	28.2%	Health care system	Model-based EE	CUA with markov model	40 years	5%	one-way sensitivity analyses and PSA	AUS$ 50,000 per QALY	*ICER (c) vs. (a): $AUS 6,346 per QALY<br>*ICER (b) vs. (c): $AUS 22,821 per QALY	Genetic testing was cost-effective
8	Panattoni L. et al. ,2012	New Zealand	ACS (included the four largest ethnic groups)	(a). clopidogrel<br>(b). prasugrel<br>(c).CYP2C19 testing guided therapy<br>Test: positive: prasugrel;<br>Test negative : clopidogrel	Pacific Island 45 %, Asian 29 %, Maori 24 %, European 15 %	Health care system	Model-based EE	CUA with decision tree model	Lifetime	3%	one-way sensitivity analyses and PSA	$NZ 50000 per QALY	*(b) is dominated by (a), high cost and lower QALYs than (a)<br>*ICER (c) vs. (a): $NZ 24,617 per QALY<br>*(b) is dominated by (c) , (c) lower cost and more effective than (b)	Genetic testing was cost-effective
9	Reese E. S. et al., 2012	USA	ACS, recent MI or stroke undergoing PCI	(a). clopidogrel<br>(b). prasugrel<br>(c). CYP2C19 testing guided therapy<br>Test: positive prasugrel;<br>Test negative clopidogrel	27% (IM or PM) 73% (UM or EM)	private payer	Model-based EE	CEA with decision tree model	15 months	5%	PSA		*(a) is dominated by (c) , (c) lower cost and more effective than (a)<br>*(b) is dominated by (c) , (c) lower cost and more effective than (b)	Genetic testing was cost-effective
**Drug: warfarin,<br>Marker: CYP2C9 and VKORC1<br>ADRs: bleeding events**														
1	Kim,DJ.. et al. ,2017	Korea	mechanical heart valve replacement (MHVR)	(a). warfarin<br>(b). CYP2C19 and VKORC1 genotyping-guided dosing of warfarin	N/A CYP2C9 wild-type/VKOR C1 AA 72.04%, CYP2C9 wild-type/VKOR C1 AG or GG 16.67%, CYP2C9 variant/VKO RC1 AA 11.29%	Health care payer (health care sector)	Model-based EE	CUA with decision tree and markov model	2 years	3%	one-way sensitivity analyses and PSA	$50,000 per QALY	*ICER (b) vs. (a): $1,356.2 per QALY	Genetic testing was cost-effective

No.	Author , Year of published	study setting	Target populations	Intervention vs comparator	Marker prevalence (%)	Perspective used	Trial- or Model-based EE	Methods	Time horizon	Discounting	Type of uncertainty analysis	WTP threshold	ICER/ICUR	Main outcome (finding)
2	Verhoef .et al. ,2016	UK and Sweden	Atrial Fibrillation (AF)	(a). warfarin<br>(b). CYP2C19 and VKORC1 genotyping-guided dosing of warfarin	N/A	National health service in UK, health-care sector in Sweden	Model-based EE	CUA with markov model	lifetime	UK 3.5 %, Sweden 3%	one-way sensitivity analyses and PSA	UK £20 000 per QALY gained, Sweden 500,000 SEK	in UK, *ICER (b) vs. (a): £6,702 per QALY in Sweden,*ICER (b) vs. (a): 253,848 SEK per QALY	Genetic testing was cost-effective
3	Mitropoulou . et al. ,2015	Croatia	ischemic stroke patients with AF	(a). warfarin<br>(b). CYP2C19 and VKORC1 genotyping-guided dosing of warfarin	N/A	healthcare system	Model-based EE	CUA with decision tree model	1 year	N/A	PSA	€40,000 to €50,000 per QALY	*ICER (b) vs. (a): €31,225 per QALY	Genetic testing was cost-effective
4	Chong, H. Y. et al. ,2014	Thailand	newly initiated warfarin therapy	(a). warfarin<br>(b). CYP2C19 and VKORC1 genotyping-guided dosing of warfarin	N/A CYP2C9*1/*1 to be 0.94 (95% CI 0.92-0.96) VKORC1 BB to be 0.05 (95% CI 0.02-0.07).	healthcare system and societal	Model-based EE	CUA with decision tree and markov model	lifetime	3%	one-way sensitivity analyses and PSA	160,000 THB or $5,333 per QALY	healthcare system perspective: *ICER (b) vs. (a):1,477,042 THB ($49,234) per QALY societal perspectiv: *ICER (b) vs. (a): 1,473,852 THB [$49,128) per QALY	Genetic testing was not cost-effective
5	You, J. H.et al. ,2014	USA	AF	(a). warfarin<br>(b). CYP2C19 and VKORC1 genotyping-guided treatment	N/A	healthcare payers	Model-based EE	CUA with decision tree and markov model	25 years	3%	PSA	$50,000 per QALY	*ICER (b) vs. (a): $ 2,843 per QALY	Genetic testing was cost-effective
6	Pink et al. ,2014	Sweden	Non-valvular AF	(a). warfarin<br>(b). CYP2C19 and VKORC1 genotyping-guided treatment<br>(c). dabigatran<br>(d). rivaroxaban<br>(e). apixaban	CYP2C9 43.9 %, VKORC1 63.5 %	National health system	Trial-based EE	CUA with discrete-event simulation model	lifetime	4%	PSA	£20,000 -30,000 per QALY	*ICER (b) vs. (a): £ 13,226 per QALY<br>*ICER (e) vs. (b): £20,671 per QALY<br>*(d) is dominated by (c) and (e), high cost and lower QALYs than (c) and €<br>*(c) is dominated by (e), high cost and lower QALYs than (e)	Genetic testing and apixaban were cost-effective

No.	Author , Year of published	study setting	Target populations	Intervention vs comparator	Marker prevalence (%)	Perspective used	Trial- or Model-based EE	Methods	Time horizon	Discounting	Type of uncertainty analysis	WTP threshold	ICER/ICUR	Main outcome (finding)
7	You et al. 2012	USA	newly diagnosed AF	(a). warfarin<br>(b). CYP2C19and VKORC1genotyping-guided treatment<br>(c). dabigatran 110 mg twice daily<br>(d). dabigatran 150 mg twice daily	N/A	Health care payer	Model-based EE	CUA with decision tree and markov model	25 years	3%	one-way,two-way sensitivity analyses and PSA	$50,000 per QALY	*(a) is dominated by (b), high cost and lower QALYs than (b)<br>*ICER (d) vs. (b): $13,810 per<br>*(c) is dominated by (d) , (d) lower cost and more effective than (c)	Genetic testing and dabigatran 150 mg was cost-effective
8	Meckley et al. 2010	USA	newly initiated warfarin therapy	(a). warfarin<br>(b). CYP2C19 and VKORC1 genotyping-guided dosing of warfarin	CYP2C9 variant 31 %, VKORC 1 variant: 30% CYP2C9 wild ('normal') 39 %	Third-party payer	Model-based EE	CUA with decision tree and markov model	lifetime	3%	one-way sensitivity analyses and PSA	$50,000 per QALY	*ICER (b) vs. (a) : $ 60,725 per QALY	Genetic testing was not cost-effective
9	Eckman et al. 2009	USA	Non-valvular AF	(a). warfarin<br>(b). CYP2C19 and VKORC1 genotyping-guided dosing of warfarin	CYP2C9 20 %, VKORC 1: 42 %	Societal	Model-based EE	CUA with markov model	lifetime	3%	one-way sensitivity analyses and PSA	$50,000 per QALY	*ICER (b) vs. (a) : $171,750 per QALY	Genetic testing was not cost-effective
10	Patrick et al. 2009	USA	newly diagnosed AF	(a). warfarin<br>(b). CYP2C19 and VKORC1 genotyping-guided dosing of warfarin	5 % ([65 years old)<br>N/A	Societal	Model-based EE	CUA with decision tree and markov model	lifetime	3%	PSA	$50,000 per QALY	*ICER (b) vs. (a) :ICER<50,000 per QALY if it increased the time spent in the target INR range during the first 3 months of treatment by 5to 9 percentage points	
11	You et al. 2009	USA	newly initiated warfarin therapy	(a). warfarin<br>(b). CYP2C19 and VKORC1 genotyping-guided dosing of warfarin	N/A	Healthcare provider	Model-based EE	CUA with decision tree	1 year	3%	PSA	$50,000 per QALY	*ICER (b) vs. (a):<br>$ 347,059 per QALY	Genetic testing was not cost-effective
12	McWilliam et al. 2008	USA	newly initiated warfarin therapy	(a). warfarin<br>(b). CYP2C19 and VKORC1 genotyping-guided dosing of warfarin	36%	No data	Trial-based EE	CEA with retrospective study	1 year	N/A	No data		*Low baseline bleeding :ICER (b) vs. (a): $82,890 per bleeding averted<br>*Medium baseline bleeding :ICER (b) vs. (a): $13,589 per bleeding averted	Genetic testing was cost-effective

No.	Author , Year of published	study setting	Target populations	Intervention vs comparator	Marker prevalence (%)	Perspective used	Trial- or Model-based EE	Methods	Time horizon	Discounting	Type of uncertainty analysis	WTP threshold	ICER/ICUR	Main outcome (finding)
													*High baseline bleeding : *(b) is dominated by (a) lower cost than (a)	
13	Schalekamp et al., 2006	The Netherlands	newly initiated warfarin therapy	(a). warfarin<br>(b). CYP2C19 and VKORC1 genotyping-guided dosing of warfarin	36%	No data	Model-based EE	CEA with decision tree model	1 year	N/A	threshold analysis	€20,000	*ICER (b) vs. (a): € 4233 per bleeding averted	Genetic testing was cost-effective
14	You et al. ,2004	USA	newly initiated warfarin therapy	(a). warfarin<br>(b). CYP2C19 and VKORC1 genotyping-guided dosing of warfarin	36%	healthcare provider	Model-based EE	CEA with decision tree model	1 year	N/A	one-way and two-way sensitivity analysis		*ICER (b) vs. (a): $5778 per bleeding averted	Genetic testing was cost-effective
**Drug: statin,<br>Marker: pharmacogenetics test,<br>ADR: myopathy, rhabdomyolysis**														
1	Mitchel . et al., 2017	Canada	cardiovascular patients	(a). statin<br>(b).genotyping-guided treatment with statin<br>(only patients experiencing musculoskeletal pain are being tested)	N/A	public payer	Model-based EE	CUA with markov model	20 years	No data	PSA, Deterministic SA	CAN $6,150 per QALY	*(a) is dominated by (b), high cost and lower QALYs than (b)<br>*(b) would be cost-effective as long as the test costs less than CAN$906.	Genetic testing was cost-effective
**Drug: allopurinol,<br>Marker: HLAB*5801,<br>ADR: SJS/TEN, DRESS**														
1	Cheng H et al. ,2018	China (Han population)	Hyperuricemia and gout	(a). allopurinol 100mg. and 600mg. per day<br>(b). febuxostat 40mg.and 80mg. per day<br>(c). HLA-B5801 testing prior to treatment<br>Test positive: febuxostat<br>Test negative: allopurinol	11.90%	No data	Trial-based EE	CMA with retrospective study	1 year	N/A	No data	-	in all 253 patients<br>*(c) saved 1,384,040.9 yuan for allopurinol and febuxostat at the lowest dosages<br>*(c) saved 2,807,770.0 yuan for allopurinol and febuxostat at the highest dosages	Genetic testing was cost-saving
2	Chong et al. ,2018	Malaysia	gout	(a). allopurinol starting dose 300mg., target dose 600mg. per day (current practice)<br>(b). probenecid target dose 2g. per day<br>(c). HLA-B5801 testing prior to treatment<br>Test positive: probenecid<br>Test negative: allopurinol	12.4%. PPV=1.5	Societal	Model-based EE	CUA with decision tree and markov model	lifetime	3%	one-way sensitivity analyses and PSA	MYR 39,000 or $8,695 per QALY	*(b) is dominated by (a), high cost and lower QALYs than (a)<br>*(c) is dominated by (a), high cost and lower QALYs than (a)	Genetic testing was not cost-effective

No.	Author , Year of published	study setting	Target populations	Intervention vs comparator	Marker prevalence (%)	Perspective used	Trial- or Model-based EE	Methods	Time horizon	Discounting	Type of uncertainty analysis	WTP threshold	ICER/ICUR	Main outcome (finding)
3	Jutkowitz et al .,2017	USA	gout	(a). allopurinol-febuxostat sequential therapy : allopurinol:target dose 300mg./day and febuxostat: 80mg./day, (current practice)<br>(b). HLA-B5801 testing prior to treatment<br>Test positive: febuxostat<br>Test negative:allopurinol	Caucasians and Hispanics 0.7%; African Americans 3.8%; and Asians 7.4%<br>PPV= (Caucasians 0.00472<br>African 0.02421<br>Asians 0.05164)	US healthcare payer	Model-based EE	CUA with decision tree and markov model	lifetime	3%	one-way,two-way sensitivity analyses and PSA	$109,000 per QALY	ICER (b) vs. (a): for Asians $64,190, African Americans $83,450, Caucasians or Hispanics $183,720 per QALY	Genetic testing was cost-effective for Asians and African Americans , but not for Caucasians or Hispanics in the US
4	Ke CH. et al., 2017	Taiwan	gout with chronic kidney disease	(a). benzbromarone 100mg/day (current practice)<br>(b). allopurinol target dose 100mg./ day<br>(c). febuxostat 80mg./day<br>(d). HLA-B5801 testing prior to treatment<br>Test positive: febuxostat or benzbromarone<br>Test negative: allopurinol	18%<br>PPV=<br>0.012<br>0.024 (CKD)	third-party payer	Model-based EE	CUA with decision tree model	1 year	N/A	one-way sensitivity analyses and PSA	NT $800,000 or US $25,600 per QALY in 2015	(b) is dominated by (a), high cost and lower QALYs than (a) current practice)<br>(c) is dominated by (d), high cost and lower QALYs than (d) current practice)<br>ICER (d) vs. (a): NT$ 234,610 per QALY in the base-case and NT$ 230,925 per QALY in patients with chronic kidney disease	Genetic testing was cost-effective
5	Plumpton CO et al., 2017	UK	gout	(a). allopurinol target dose 300mg. per day add prophylactic treatment with colchicine<br>(b). HLA-B5801 testing prior to treatment<br>Test positive: febuxostat : target dose 80mg. per day<br>Test negative: allopurinol	European : 1.13%<br>PPV SJS/TEN = 0.0013<br>PPV DRESS = 0.0067	National Health Service	Model-based EE	CUA with decision tree and markov model	lifetime	3.5 %	one-way sensitivity analyses and PSA<br>Scenario analysis	£30,000 per QALY	ICER (d) vs. (a): £44,954 in the base case and £38,478 per QALY in patients with chronic renal insufficiency	Genetic testing was not cost-effective

No.	Author , Year of published	study setting	Target populations	Intervention vs comparator	Marker prevalence (%)	Perspective used	Trial- or Model-based EE	Methods	Time horizon	Discounting	Type of uncertainty analysis	WTP threshold	ICER/ICUR	Main outcome (finding)
6	Dong D et al., 2015	Singapore	gout	(a). allopurinol starting dose 300mg., target dose 600mg. per day<br>(b). allopurinol +safety program (SP)<br>(c). HLA-B5801 testing prior to treatment + SP<br>(d). HLA-B5801 testing prior to treatment + SP<br>Test positive: probenecid target dose 2g. per day<br>Test negative: allopurinol<br>(e). HLA-B5801 testing prior to treatment<br>Test positive: probenecid target dose 2g. per day<br>Test negative: allopurinol<br>(f). no allopurinol (treatment of acute flares only)	18.5% PPV=1.52%	health systems	Model-based EE	CUA with decision tree model	lifetime	3%	one-way sensitivity analyses and PSA	$50,000 per QALY	*ICER (b) vs. (a): $79,140 per QALY<br>*(c) is dominated by (b), high cost and lower QALYs than (b) current practice)<br>*ICER (d) vs. (b): $85,630 per QALY<br>*(e) is dominated by (d), high cost and lower QALYs than (d) current practice)<br>*(f) is dominated by (d), high cost and lower QALYs than (d) current practice)	Genetic testing or safety programs was not cost-effective.
7	Park DJ et al., 2015	Korea	gout with chronic renal insufficiency	(a). allopurinol starting dose 100mg., target dose 300mg. per day<br>(b). HLA-B5801 testing prior to treatment<br>Test positive: febuxostat starting dose 40mg., target dose 80mg.) per day<br>Test negative: allopurinol	12.2% PPV=0.18	National health payer	Model-based EE	CBA with decision tree model	1 year	N/A	one-way sensitivity analyses		costs of (a) $1,193 and (b) $1,055<br>(b) is less costly and more effective than (a)	Genetic testing was cost-saving
8	Saokaew et al., 2014	Thailand	gout	(a). allopurinol starting dose 300mg per day<br>(b). HLA-B5801 testing prior to treatment<br>Test positive: probenecid target dose 1mg target to 2g. per day<br>Test negative: allopurinol	15% PPV=0.01049	Societal	Model-based EE	CUA with decision tree and markov model	life time	3%	one-way, scenario and PSA	160,000 THB per QALY	*ICER (b) vs. (a): 156,937 THB per QALY	Genetic testing was cost-effective

No.	Author , Year of published	study setting	Target popula tions	Intervention vs comparator	Marker prevalence (%)	Perspe ctive used	Trial- or Model-based EE	Methods	Time horizon	Disc oun ting	Type of uncertainty analysis	WTP threshold	ICER/ICUR	Main outcome (finding)
**Drug: abacavir, Marker: HLA B*57:01, ADR: hypersensitivity**														
1	Kubaeva et al. ,2018	Russia	HIV	(a). abacavir regimen<br>(b). HLA-B* 5701 testing prior to treatment<br>Test positive: alternative regimens without abacavir<br>Test negative: abacavir regimen	N/A	Health care system	Trial-based EE	CMA with retrospective study	N/A	N/A	No data		(b) vs. (a): was cost-saving 54,164.6 rubles	Genetic testing was cost-saving
2	Kapoor R et al., 2015	3 ethnic groups in Singap ore :	HIV	(a). abacavir-based ART<br>(b).tenofovir-based ART<br>(c).HLA-B:5701 testing prior to treatment<br>Test positive: tenofovir-based ART<br>Test negative: abacavir-based ART	Han Chinese: 1.1 %<br>Southeast Asian Malay: 1.8 %<br>South Asian Indian: 6.3 %<br>PPV= 0.67,1.1,3.86<br>PPV for clinically suspected cases 61.20%	No data	Model-based EE	CUA with decision tree model	lifetime	No data	one-way sensitivity analyses and PSA	$50,000 per QALY	**Early stage:** ICER (c) vs. (a):<br>Chinese $415,845 per QALY,<br>Malay $318,029 per QALY,<br>Indian $208,231 per QALY<br>**Late stage:** ICER (c) vs. (a):<br>Chinese $926,938 per QALY,<br>Malay $624,297 per QALY,<br>Indian $284,598 per QALY<br>**HIV who are contraindicated to tenofovir**<br>ICER (b) vs. (a): all ethnicity was not cost-effective , except for Indian patients with early-stage ICER (c) vs. (a): $44,649 per QALY	Genetic testing was not cost-effective, except for Indian patients with early-stage HIV who are contraindic ated to tenofovir
3	Calatrava et al., 2010	Spain	HIV	(a). abacavir regimen<br>(b). HLA-B:5701 testing prior to treatment<br>Test positive: alternative HAART regimen without ABC<br>Test negative: abacavir regimen	5.6%<br>PPV= 0.612	National health system	Model-based EE	CEA with decision tree model	6 months	N/A	Univariate, Bivariate, sensitivity analyses and PSA		(b) is dominated by (a), high cost and lower benefit than (a)	Genetic testing was not cost-effective

No.	Author , Year of published	study setting	Target populations	Intervention vs comparator	Marker prevalence (%)	Perspective used	Trial- or Model-based EE	Methods	Time horizon	Discounting	Type of uncertainty analysis	WTP threshold	ICER/ICUR	Main outcome (finding)
4	Kauf TL et al., 2010	USA	HIV	(a). short-term: abacavir+ lamivudine+efavirenz.<br>(b).long-term: tenofovir+emtricitabine+efavirenz<br>(c). HLA-B:5701 testing prior to treatment<br>Test positive: tenofovir+emtricitabine+efavirenz.<br>Test negative: abacavir+lamivudine+efavirenz;	5.66%<br>PPV= N/A	Health care system	Model-based EE	CUA with decision tree model	lifetime	3%	one-way sensitivity and scenario analyses	$50,000 per QALY	*short-term: (c) is dominated by (a), high cost and lower QALYs than (a)<br>*long-term: (b) is dominated by (c), high cost and lower QALYs than (c)	Genetic testing was not cost-effective
5	Wolf et al. 2010	Germany	HIV	(a).combination of abacavir+ lamivudine<br>(b). HLA-B:5701 testing prior to treatment<br>Test positive combination of tenofovir, emtricitabine<br>Test negative combination of abacavir+ lamivudine	7.3%<br>PPV= N/A	Societal and healthcare payer,	Model-based EE	CBA with decision tree model	six weeks	N/A	one-way and multi-way sensitivity analyses		(b) vs. (a): (b) cost-saving €44 and 127 per screened patient from healthcare payer and societal perspective	Genetic testing was cost-saving
6	Schackman BR et al., 2008	USA	HIV	(a). abacavir-based regimen<br>(b).tenofovir-based regimen<br>(c).HLA-B:5701 testing prior to treatment<br>Test positive: tenofovir or AZT-based regimen<br>Test negative: abacavir-based regimen	5.7%<br>PPV= 0.479	health system Medicaid programs	Model-based EE	CUA with decision tree model	lifetime	3%	one-way sensitivity analyses	$50,000 per QALY	*ICER (c) vs. (a): $36,700 per QALY<br>*(b) is dominated by (a) ,high cost and lower QALYs than (a)	Genetic testing was cost-effective
7	Hughes DA et al., 2004	UK	HIV	(a).Trizivir (AZT/3TC/ABC)<br>(b). HLA-B:5701 testing prior to treatment<br>Test positive: HAART regimen without abacavir<br>Test negative: Trizivir (AZT/3TC/ABC)<br>(HAART: highly active antiretroviral therapy)	3.7%<br>PPV= 0.16	National health system	Model-based EE	CEA with decision tree model	6 months	N/A	Multiple univariate sensitivity, threshold analysis, PSA		(a) is dominated by (b) ,high cost and lower benefit than (b), except for Trizivir is substituted with ritonavir+indinavir+combivir ICER (b) vs. (a): € 22,811 per hypersensitivity reaction avoided	Genetic testing was cost-effective to ICER € 22 811 per hypersensitivity reaction avoided.

No.	Author , Year of published	study setting	Target populations	Intervention vs comparator	Marker prevalence (%)	Perspective used	Trial- or Model-based EE	Methods	Time horizon	Discounting	Type of uncertainty analysis	WTP threshold	ICER/ICUR	Main outcome (finding)
**Drug: efavirenz,<br>Marker: CYP2B6,<br>ADRs: sub- or supratherapeutically dosed**														
1	Schackman. et al., 2015	USA	HIV	(a). efavirenz 600 mg+tenofovir+ emtricitabine<br>(b). CYP 2B6 testing prior to treatment<br>Test positive: decrease dose to 200,400 mg<br>Test negative: efavirenz 600 mg<br>(c). Universal low dose efavirenz 400 mg +tenofovir+ emtricitabine	39.7% (600 mg) 47.1% (400 mg) 13.2% (200 mg) genotyping is 99.7% conclusive	Health care payer Health system	Model-based EE	CUA with decision tree model	Lifetime	3%	one-way sensitivity analyses and threshold analyses	$ <100,000 per QALY	(a) is dominated by (b) ,high cost and lower benefit than (b)	Genetic testing was cost-effective
**Drug: azathioprine,<br>Marker: TPMT<br>ADR: neutropenia or severe neutropenia**														
1	Thompson AJ et al., 2014.	UK	autoimmune diseases	(a). azathioprine therapy<br>(b). TPMT testing prior to treatment<br>Test positive: alternative treatment<br>Test negative: azathioprine	N/A	health service	Trial-based EE	CUA with a prospective Trial based evaluation	4 months	N/A	one-way sensitivity analyses and bootstrap	£20,000 per QALY	*(b) is dominated by (a) lower cost and lower QALYs than (a)	Genetic testing was not cost-effective
2	Priest VL et al., 2006	New Zealand	inflammatory bowel disease (IBD)	(a). azathioprine<br>(b). TPMT testing prior to treatment (Phenotype and genotype)	0.60%	payer's perspective	Model-based EE	CUA with decision tree model	1 year	N/A	Univariate sensitivity analyses		Phenotype and genotype testing dominated by (a), high cost and lower QALYs than (a). There are cost-savings (vs no testing) of $NZ120,000 and 71 neutropenias avoided;-0.34QALYs and $NZ11,000 and 40 neutropenias avoided, -0.58QALYs)	Genetic testing was not cost-effective
**Drug: azathioprine,<br>Marker: TPMT<br>ADR: leukopenia**														
3	Hagaman JT et al., 2010	USA.	Idiopathic Pulmonary Fibrosis (IPF)	(a). conservative therapy which no specific therapy<br>(b). azathioprine+N-acetylcysteine and steroids (no testing)<br>(c). TPMT testing prior to	12.5 Normal (high): 87.6%, Intermediate:	No data	Model-based EE	CUA with decision tree model	1 year	N/A	one way and two way sensitivity analysis	$50,000 per QALY	*ICER (b) vs. (a): $49,245 per QALY<br>*ICER (c) vs. (b): $29,663 per QALY	Genetic testing was cost-effective

No.	Author , Year of published	study setting	Target populations	Intervention vs comparator	Marker prevalence (%)	Perspective used	Trial- or Model-based EE	Methods	Time horizon	Discounting	Type of uncertainty analysis	WTP threshold	ICER/ICUR	Main outcome (finding)
				treatment<br>Test positive: conservative therapy<br>Test negative: azathioprine+N-acetylcysteine and steroid	11.9%, Low: 0.5%									
4	Dubinsky MC et al., 2005	USA	Crohn's disease	(a). TPMT testing prior to treatment<br>Test positive: metrotrexate<br>Test negative: azathioprine<br>(b). metabolite monitoring (MM) prior to treatment with azathioprin<br>(c). TPMT testing +MM prior to treatment with azathioprine<br>(d). community care (CC)	N/A	third-party payer perspective	Model-based EE	CEA with decision tree model	1 year	N/A	One-way , 2-way sensitivity analysis		*(d) is dominated by (a), (b),and (c),<br>(d) less costs and faster time to response or sustained response	Genetic testing was cost-effective
5	Winter J et al., 2004	Scotland	IBD	(a). azathioprine<br>(b). TPMT testing prior to treatment	N/A<br>PPV = 9%;	payer's perspective (NHS)	Trial-based EE	CEA with case based model	N/A	N/A	No data		*for a 30 year old: ICER (b) vs. (a): £347 per life-year saved and<br>* for a 60 year old.: ICER (b) vs. (a): £817 per life-year saved	Genetic testing was cost-effective
**Drug: azathioprine,<br>Marker: TPMT<br>ADR: all adverse events**														
6	Sayani FA et al., 2005	Canada	Crohn's disease and IBD	(a). azathioprine<br>(b). TPMT testing prior to treatment	N/A	No data	Trial-based EE	CMA with a randomized prospective study	1 year	N/A	No data		The direct health care costs for (a) $300.11 per patient and (b) $348.87 per patient	Genetic testing was not cost-saving
				**Drug: azathioprine, Marker: TPMT<br>ADR: severe bone marrow toxicity**										
7	Oh KT et al., 2004	Korea	rheumatoid arthritis and systemic lupus erythematosus	(a). azathioprine<br>(b). TPMT testing prior to treatment	11.3%	societal	Model-based EE	CEA with decision tree model	1 year	N/A	One-way sensitivity analysis		*(a) is dominated by (b), (b) lower cost snd more effective than (a)	Genetic testing was cost-effective
				**Drug: azathioprine, Marker: TPMT<br>ADR: hematological cytopenias**										

No.	Author , Year of published	study setting	Target populations	Intervention vs comparator	Marker prevalence (%)	Perspective used	Trial- or Model-based EE	Methods	Time horizon	Discounting	Type of uncertainty analysis	WTP threshold	ICER/ICUR	Main outcome (finding)
8	Marra CA et al., 2002.	Canada	rheumatoid arthritis and systemic lupus erythematosus	(a). azathioprine<br>(b). TPMT testing prior to treatment	N/A	third party payer perspective	Model-based EE	CBA with decision tree model	6 months	N/A	One-way sensitivity analysis		(a) cost $677 Cdn per patient, (b) cost $663 Cdn per patient.	Genetic testing was cost-saving
**Drug: carbamazepine, Marker: HLAB*15:02, ADRs: SJS, TEN**														
1	Chong. et al.,2017	Malaysia	Epilepsy	(a). carbamazepine (current practice)<br>(b). sodium valproate (VPA)<br>(c). HLA-B*15:02 testing prior to treatment<br>Test positive: VPA<br>Test negative: carbamazepine	15%<br>PPV= 2.951	societal	Model-based EE	CUA with decision tree and markov model	lifetime	3%	one-way sensitivity analyses and PSA	MYR 37,000 ($ 8982) per QALY	*(b) is dominated by (a), high cost and lower QALYs than (a) current practice)<br>*(c) is dominated by (a), high cost and lower QALYs than (a) current practice)	Genetic testing was not cost-effective
2	Chen et al.,2016	Hong Kong	Epilepsy	(a). current situation, using antiepileptic drug (pre-policy period)<br>(b). current situation, using antiepileptic drug (post-policy period)<br>(c). HLA-B*15:02 testing prior to treatment (the ideal situation) (ideal situation)<br>Test positive: alternative anti-epileptic drug<br>Test negative: carbamazepine or phenytoin<br>(d). HLA-B*15:02 testing prior to either carbamazepine or phenytoin (extended situation)	16.8%<br>PPV= CBZ-SJS: 6.69% for carriers and 0.07% for non-carriers	Health care provider	Model-based EE	CUA with decision tree model	1 year	N/A	one-way sensitivity analyses and PSA	$50,000 per QALY	*ICER (b) vs. (a): $85,697 per QALY<br>*ICER (c) vs. (a): $11,090 per QALY<br>*ICER (d) vs. (a): $197,158 per QALY	Genetic testing was cost-effective
3	Rattanavipapong W. et al., 2013	Thailand.	Epilepsy or neuropathic pain	(a). carbamazepine (no HLA-B*15:02 testing)<br>(b). HLA-B*15:02 testing prior to treatment<br>Test positive:<br>for epilepsy :valproate,<br>for neuropathic pain : gabapentin;<br>Test negative: carbamazepine<br>(c). all prescribed alternative (no HLA-B*15:02 testing)	15.5%<br>PPV= 0.019	Societal	Model-based EE	CUA with decision tree and markov model	lifetime	3%	one-way sensitivity analyses and PSA	120,000 THB per QALY	*ICER (b) vs. (a): 222,000 THB per QALY for epilepsy, 130,000 THB per QALY for neuropathic pain<br>*ICER (c) vs. (a): 32,522,000 per QALY for epilepsy, (epilepsy), 35,877,000 per QALY for neuropathic pain	Genetic testing was not cost-effective

No.	Author , Year of published	study setting	Target populations	Intervention vs comparator	Marker prevalence (%)	Perspective used	Trial- or Model-based EE	Methods	Time horizon	Discounting	Type of uncertainty analysis	WTP threshold	ICER/ICUR	Main outcome (finding)
				(epilepsy : valproate,<br>neuropathic pain : gabapentin)										
4	Tiamkao S. et al., 2013	Thailand	Epilepsy or neuropathic pain and neurological diseases	(a). carbamazepine<br>(b). HLA-B*15:02 testing prior to treatment<br>Test positive: non-specified.<br>Test negative: carbamazepine	8.4%<br>PPV= 7.4	No data	Trial-based EE	CBA with retrospective study	N/A	N/A	No data		(b) vs. (a): (b) was cost-saving 98,549.94 THB per 100 cases of carbamazepine users	Genetic testing was cost-saving
5	Dong D. et al., 2012	Singapore	Epilepsy	(a).carbamazepine or phenytoin<br>(b). HLA-B*15:02 testing prior to treatment<br>Test positive :valproate;<br>Test negative carbamazepine or phenytoin<br>(c).valproate (no screening)	14.87%<br>PPV=5.96	Healthcare provider (National health system)	Model-based EE	CUA with decision tree model	30 year	3%	one-way sensitivity analyses and PSA	$50,000 per QALY	*ICER (b) vs. (a): Chinese patients $37,030 per QALY, Malay $7,930 per QALY and Indians $136,630 per QALY<br>*(c) is dominated by (b), high cost and lower QALYs than (b)	Genetic testing was cost-effective for Chinese and Malays but not for Indians in Singapore.
**Drug: carbamazepine,<br>Marker: HLAA*31:01,<br>ADRs: SJS, TENs, hypersensitivity**														
1	Plumpton et al. 2015	UK	Epilepsy	(a). carbamazepine<br>(b). HLA-B*31:01 testing prior to treatment<br>Test positive : lamotrigine;<br>Test negative : carbamazepine	European : 2.59 %	National health system	Model-based EE	CUA with decision tree and markov model	lifetime	3.50 %	one-way sensitivity analyses and PSA	£20,000 per QALY	*ICER (b) vs. (a): £ 12,808 per QALY	Genetic testing was cost-effective
**Drug: Fluoropyrimidines,<br>Marker: DPYD*2A genotype–guided dosing.<br>ADR: toxicity i.e.hematologic, GI**														
1	Deenen MJ et al., 2016	Netherland	cancer	(a). fluoropyrimidines-based therapy<br>(b). DPYD*2A testing prior to treatment<br>Test positive: alternative regimen<br>Test negative: Fluoropyrimidines-based	98.8%<br>22 patients (1.1%) proved to be heterozygously polymorphic for DPYD*2A	healthcare payer	Model-based EE	CBA with decision tree model and cohort study	N/A	N/A	one-way sensitivity analyses and PSA		*(b) vs. (a): (b) cost-savings of €45 ($61) per patient	Genetic testing was cost-saving

No.	Author , Year of published	study setting	Target populations	Intervention vs comparator	Marker prevalence (%)	Perspective used	Trial- or Model-based EE	Methods	Time horizon	Discounting	Type of uncertainty analysis	WTP threshold	ICER/ICUR	Main outcome (finding)
**Drug: irinotecan,<br>Marker: UGT1A1<br>ADR: severe neutropenia**														
1	Picherea u S et al., 2010.	France	metastatic colorectal cancer	(a).FOLFIRI regimen (5-fluorouracil, leucovorin and irinotecan)<br>(b). UGT1A1 testing prior to treatment with FOLFIRI<br>Test positive: FOLFOX regimen (oxaliplatine + 5-FU + folinic acid)<br>Test negative: FOLFIRI	G6/6: 42.5%<br>G6/7: 47.9%<br>G7/7: 9.6%	hospital perspective	Model-based EE	CEA with decision tree model	28 days	N/A	one-way sensitivity analyses		*ICER (b) vs. (a): to avoid one febrile neutropenia per 1000 patients treated was € 942.8 to € 1090.1.	Genetic testing was cost-effective
2	Gold HT et al., 2009	USA	metastatic colorectal cancer	(a).FOLFIRI regimen (5-fluorouracil, leucovorin and irinotecan)<br>(b). UGT1A1 testing prior to treatment with FOLFIRI<br>Test positive: reduce dose to intermediate dose<br>Test negative: FOLFIRI	11%	Medicare payer	Model-based EE	CUA with decision tree model	5 years	3%	one-way sensitivity analyses and PSA	$100,000 per QALY	*ICER (b) vs. (a): was cost-effective if the treatment efficacy of irinotecan in homozygotes after dose reduction had to be ≥ 98.4% of full-dose efficacy for genetic testing to remain preferred	Genetic testing was cost-effective
**Drug: Nortriptyline,<br>Marker: CYP2D6<br>ADRs: sub- or supratherapeutically dosed**														
1	Bern EJ. et al., 2016	The Netherland	major depressive disorder	(a). nortriptyline<br>(b). CYP2D6 genotyping-guided dosing of nortriptyline	1.Poor metabolize 8%<br>2.Intermediate metabolizer 11%<br>3.Extensive metabolizer 79%<br>4.Ultra-rapid metabolizer 2%	health-care insurance payers	Model-based EE	CUA with decision tree model	12 weeks	N/A	one-way sensitivity analyses and PSA	€50 000 per QALY	*ICER (b) vs. (a): €1,333,000 per QALY	Genetic testing was not cost-effective
**Drug: oral contraceptive,<br>Marker: factor V Leiden<br>ADR: Thromboembolism**														

No.	Author , Year of published	study setting	Target populations	Intervention vs comparator	Marker prevalence (%)	Perspective used	Trial- or Model-based EE	Methods	Time horizon	Discounting	Type of uncertainty analysis	WTP threshold	ICER/ICUR	Main outcome (finding)
1	Smith KJ, 2008	USA	women who receiving oral contraceptives (OCPs)	(a). usual care<br>(b). genotyping<br>(c).genotyping with OCP counseling<br>(d). genotyping with OCP counseling and AC for high-risk events<br>(e). genotyping with OCP counseling and AC for long-term	0.1-3.9%	No data	Model-based EE	CUA with decision tree model	30 years	3%	PSA	$20,000 per QALY	*ICER (d) vs. (c): $ 147 per QALY<br>*ICER (e) vs. (d): $ 639,500 per QALY<br>*(a) is dominated by (d), high cost and lower QALYs than (d)<br>*(b) is dominated by (d), high cost and lower QALYs than (d))	Genetic testing, counselling and prophylactic AC during high-risk periods in female relatives of FVL carriersw as cost-effective

AC: anticoagulation, **ACS:** Acute Coronary Syndrome, **CBA:** Cost-benefit analysis, **CEA:** Cost-effectiveness analysis, **CMA:** Cost-minimization analysis, **CUA:** Cost-utility analysis, **EE:** Economic evaluation, **ICER:** Incremental Cost-Effectiveness Ratio
OCP: oral contraceptive pill, **PCI:** Percutaneous Coronary Intervention, **PSA:** Probabilistic Sensitivity Analysis
QALY: Quality adjusted life-year, **N/A:** Not Applicable, **WTP:** Willingness to pay

Table A3. The assessment of quality of reporting using CHEERS checklist [14]

Author/ Year	1	2	3	4	5	6	7	8	9	10	11 a	11 b	12	13 a	13 b	14	15	16	17	18	19	20 a	20 b	21	22	23	24
**Drug: allopurinol Marker: HLAB* 5801 ADR: hypersensitivity**																											
Cheng H et al.,2018	1	0.5	1	1	1	0.5	1	1	N/A	1	1	N/A	1	1	N/A	1	N/A	N/A	0	0	1	0	N/A	1	1	0	1
Chong et al.,2018	1	1	1	1	1	1	1	1	1	1	1	N/A	1	N/A	1	1	1	1	1	1	1	N/A	1	1	1	0	1
Jutkowitz et al.,2017	1	1	1	1	1	1	1	1	1	1	N/A	1	1	N/A	1	1	1	1	1	1	1	N/A	1	1	1	1	1
Ke CH. et al., 2017	1	1	1	1	1	1	1	1	N/A	1	1	N/A	1	N/A	1	1	1	1	1	1	1	N/A	1	1	1	1	0
Plumpton et al., 2017	1	1	1	1	1	1	1	1	1	1	N/A	1	1	N/A	1	1	1	1	1	1	1	N/A	1	1	1	1	1
Dong D et al., 2015	1	0.5	1	1	1	1	1	1	1	1	1	N/A	1	N/A	1	1	1	1	1	1	1	N/A	1	1	1	1	1
Park DJ et al., 2015	1	1	1	1	1	1	1	1	N/A	1	1	N/A	1	N/A	1	0.5	1	1	1	1	1	N/A	1	1	1	1	0
Saokaew S.et al., 2014	1	1	1	1	1	1	1	1	1	1	1	N/A	1	N/A	1	1	1	1	1	1	1	N/A	1	0	1	1	1
**Drug: abacavir Marker: HLA B*57:01 ADR: hypersensitivity**																											
Kubaeva et al.,2018	1	0.5	1	1	0	1	0	0	0	1	1	N/A	1	1	N/A	0	N/A	N/A	0	0	1	0	N/A	0	0.5	0	1
Kapoor R et al., 2015	1	0.5	1	1	1	0.5	1	1	0	1	1	N/A	1	N/A	1	0.5	1	1	1	1	1	N/A	1	1	1	1	1
Calatrava et al., 2010	1	1	1	1	1	1	1	1	N/A	1	1	N/A	1	N/A	1	1	1	1	1	1	1	N/A	1	1	1	0	1
Kauf TL et al., 2010	1	1	1	1	1	1	1	1	1	1	1	N/A	1	N/A	1	1	1	1	1	1	1	N/A	1	1	1	1	0
Wolf et al. 2010	0.5	0.5	1	1	1	1	1	1	N/A	1	1	N/A	1	N/A	1	1	1	1	1	1	1	N/A	1	0	1	1	1
Schackman et.al.2008	1	1	1	1	1	1	1	1	1	1	1	N/A	1	N/A	1	1	1	1	1	1	1	N/A	1	1	1	1	1
Hughes et al., 2004	1	1	1	1	1	1	1	1	N/A	1	N/A	1	1	N/A	1	1	1	1	1	1	1	N/A	1	1	1	0	0
**Drug: carbamazepine Marker: HLAB* 15:02 ADRs: SJS, TEN**																											
Chong. et al.,2017	1	1	1	1	1	1	1	1	1	1	N/A	1	1	N/A	1	1	1	1	1	1	1	N/A	1	1	1	1	1
Chen et al.,2016	1	0.5	1	1	1	1	1	1	N/A	1	1	N/A	1	N/A	1	1	1	1	1	1	1	N/A	1	0	1	1	1

Author/ Year	1	2	3	4	5	6	7	8	9	10	11 a	11 b	12	13 a	13 b	14	15	16	17	18	19	20 a	20 b	21	22	23	24
Rattanavipapong W. et al., 2013	1	0.5	1	1	1	1	1	1	1	1	1	N/A	1	N/A	1	1	1	1	1	1	1	N/A	1	1	1	1	1
Tiamkao S. et al., 2013	1	0.5	1	1	1	0.5	1	0	0	1	1	N/A	1	1	N/A	0	N/A	N/A	0	1	1	0	N/A	0	1	1	1
Dong D. et al., 2012	1	0.5	1	1	1	1	1	1	1	1	1	N/A	1	N/A	1	1	1	1	1	1	1	N/A	1	1	1	1	1
**Drug: carbamazepine Marker: HLAA* 31:01 ADRs: SJS, TENs, hypersensitivity**																											
Plumpton et al. 2015	1	1	1	1	1	1	1	1	1	1	1	N/A	1	N/A	1	1	1	1	1	1	1	N/A	1	1	1	1	1
**Drug: clopidogrel Marker: CYP2C19 ADRs: major cardiac/adverse CV events**																											
Wang Y. et al.,2018	1	1	1	1	1	1	1	1	1	1	1	N/A	1	N/A	1	1	1	1	1	1	1	N/A	1	0	1	1	1
Jiang, M. et al.,2017	1	1	1	1	0	1	1	1	1	1	1	N/A	1	N/A	1	1	1	1	1	1	1	N/A	1	0	1	1	1
Deiman BA et al.,2016	1	0.5	1	1	1	0.5	1	0	0	0	1	N/A	1	1	N/A	0	N/A	N/A	0	0	1	0		0	1	1	1
Kazi D.S. et al.,2014	1	1	1	1	1	1	1	1	1	1		1	1	N/A	1	1	1	1	1	1	1	N/A	1	1	1	1	1
Patel et al.,2014	1	1	1	1	0	1	1	1	1	1	1	N/A	1	N/A	1	1	1	1	1	1	1	N/A	1	0	1	1	1
LALA A. et al., 2013	1	0.5	1	1	1	1	1	1	1	1	1	N/A	1	N/A	1	1	1	1	1	1	1	N/A	1	0	1	1	1
Zorich et al., 2013	1	0.5	1	1	1	1	1	1	1	1	1	N/A	1	N/A	1	1	1	1	1	1	1	N/A	1	0	1	1	1
Panattoni L et al.,2012	1	1	1	1	1	1	1	1	1	1	1	N/A	1	N/A	1	1	0	0	1	1	1	N/A	1	1	1	1	1
Reese E. S. et al., 2012	1	0.5	1	1	1	1	1	1	1	1	1	N/A	1	N/A	1	1	1	1	1	1	0.5	N/A	1	0	1	1	0
**Drug: warfarin Marker: CYP2C9 and VKORC1 ADRs: bleeding events**																											
Kim, D. J..et al.2017	1	1	1	1	1	1	1	1	1	1	1	N/A	1	N/A	1	1	1	1	1	1	1	N/A	1	0	1	1	1
Verhoef .et al.2016	1	1	1	1	1	1	1	1	1	1	1	N/A	1	N/A	1	1	1	1	1	1	1	N/A	1	1	1	1	1
Mitropoulou.et al.2015	1	0.5	1	1	1	1	1	1	N/A	1	1	N/A	1	N/A	1	0	1	1	1	1	1	N/A	1	0	1	1	1
Chong, H. et al.2014	1	0.5	1	1	1	1	1	1	1	1	N/A	1	1	N/A	1	1	1	1	1	1	1	N/A	1	0	1	0	1
You, J. H.et al.2014	1	0.5	1	1	1	1	1	1	1	1	1	N/A	1	N/A	1	1	1	1	1	1	1	N/A	1	0	1	1	1

Author/ Year	1	2	3	4	5	6	7	8	9	10	11 a	11 b	12	13 a	13 b	14	15	16	17	18	19	20 a	20 b	21	22	23	24
Pink et al.2014	1	0.5	1	1	1	1	1	1	1	1	N/A	1	1	1	N/A	1	N/A	N/A	1	1	1	1	N/A	1	1	1	1
You et al. 2012	1	0.5	1	1	1	1	1	1	1	1	1	N/A	1	N/A	1	1	1	1	1	1	1	N/A	1	0	1	1	1
Meckley et al. 2010	1	1	1	1	1	1	1	1	1	1	1	N/A	1	N/A	1	1	1	1	1	1	1	N/A	1	0	1	1	1
Eckman et al. 2009	1	1	1	1	1	1	1	1	1	1	N/A	1	1	N/A	1	1	1	1	1	1	1	N/A	1	0	1	1	1
Patrick et al. 2009	1	1	1	1	1	1	1	1	1	1	1	N/A	1	N/A	1	1	1	1	1	1	1	N/A	1	0	1	1	1
You et al. 2009	1	1	1	1	1	1	1	1	1	1	1	N/A	1	N/A	1	1	1	1	1	1	1	N/A	1	1	1	1	1
McWilliam et al. 2008	0	0.5	1	1	0	0.5	1	1	N/A	1	N/A	1	1	1	N/A	0.5	N/A	N/A	0	1	1	0	N/A	0	1	1	1
Schalekamp et al.2006	1	0.5	1	1	0	0	1	1	N/A	1	1	N/A	1	N/A	1	1	1	1	1	1	1	N/A	1	1	1	0	1
You et al. 2004	1	1	1	1	1	1	1	1	N/A	1	1	N/A	1	N/A	1	0	1	1	1	1	1	N/A	1	1	1	1	0
**Drug: Nortriptyline Marker: CYP2D6 sub- or supratherapeutically dosed**																											
Bern EJ. et al., 2015	1	1	1	1	1	1	1	1	N/A	1	1	N/A	1	N/A	1	1	1	1	1	1	1	N/A	1	1	1	1	1
**Drug: efavirenz Marker: CYP2B6 sub- or supratherapeutically dosed**																											
Schackman.et al., 2015	1	0.5	1	1	1	1	1	1	1	1	1	N/A	1	N/A	1	1	1	1	1	1	1	N/A	1	1	1	1	1
**Drug: statin therapy Marker:genotype screening. ADR: Myopathy**																											
Mitchel. et al., 2017	1	1	1	1	1	1	1	1	0	1	1	N/A	1	N/A	1	1	1	1	1	1	1	N/A	1	1	1	1	1
**Drug: Fluoropyrimidines Marker: DPYD*2A genotype-guided dosing. ADR: toxicity i.e.hematologic, GI**																											
Deenen et al., 2016	0.5	0.5	1	1	1	1	1	0	0	1	1	N/A	1	N/A	1	1	1	1	1	0.5	1	N/A	1	0	1	1	1
**Drug: azathioprine Marker: TPMT ADR: severe neutropenia**																											
Thompson, AJ et al., 2014.	1	0.5	1	1	1	1	1	1	N/A	1	1	N/A	1	1	N/A	1	N/A	N/A	1	1	1	1	N/A	0	1	1	1
Hagaman JTet al., 2010	1	1	1	1	0	0.5	1	1	N/A	1	1	N/A	1	N/A	1	1	1	1	1	1	1	N/A	1	0	1	0	0
Priest VLet al., 2006	1	0.5	1	1	1	1	1	1	N/A	1	1	N/A	1	N/A	1	1	1	1	1	0	1	N/A	1	1	1	1	1
Sayani FA et al., 2005	0.5	0.5	1	1	1	0.5	1	1	N/A	0	1	N/A	1	1	N/A	0	N/A	N/A	0	1	1	0	N/A	0	1	0	0

Author/ Year	1	2	3	4	5	6	7	8	9	10	11 a	11 b	12	13 a	13 b	14	15	16	17	18	19	20 a	20 b	21	22	23	24
Dubinsky et al., 2005	1	1	1	1	0	1	1	1	N/A	1	1	N/A	1	N/A	1	1	1	1	1	0.5	1	N/A	1	0	1	0	0
Winter J et al., 2004	1	0.5	1	1	0	1	1	0	0	1	1	N/A	1	1	N/A	0	N/A	N/A	0	0.5	1	0	N/A	0	1	0	0
Oh KT et al., 2004	1	1	1	1	1	1	1	1	N/A	1	1	N/A	1	N/A	1	1	1	1	1	0.5	1	N/A	1	0	1	0	1
Marra CA et al., 2002.	1	1	1	1	1	1	1	1	N/A	1	1	N/A	1	N/A	1	1	1	1	1	1	0.5	N/A	1	0	1	1	0
**Drug: irinotecan Marker: UGT1A1 ADR: severe Neutropenia**																											
Pichereau et al., 2010.	1	1	1	1	1	1	1	1	N/A	1	1	N/A	1	N/A	1	1	1	1	1	0.5	1	N/A	1	0	1	1	0
Gold HT et al., 2009	1	1	1	1	1	1	1	1	1	1	1	N/A	1	N/A	1	1	1	1	1	1	1	N/A	1	1	1	1	1
**Drug: oral contraceptive Marker: factor V Leiden ADR: Thromboembolism**																											
Smith KJ, 2008	0.5	0.5	1	1	0	0.5	1	1	1	1	1	N/A	1	N/A	1	0	1	1	1	1	1	N/A	1	0	1	1	0
**No. of study met criteria (1)**	54	32	59	59	50	50	58	54	**32**	57	50	9	59	9	50	48	**49**	**49**	52	50	57	2	50	28	58	47	46
**No. of study met criteria but not complete (0.5)**	4	27	0	0	0	9	0	0	0	0	0	0	0	0	0	3	0	0	0	5	2	0	0	0	1	0	0
**No. of study missed criteria (0)**	1	0	0	0	9	0	1	5	7	2	0	0	0	0	0	8	1	1	7	4	0	7	0	31	0	12	13
**Data not applicable (N/A)**	0	0	0	0	0	0	0	0	**20**	0	0	0	0	0	0	0	**9**	**9**	0	0	0	0	0	0	0	0	0
**Total**	**59**	**59**	**59**	**59**	**59**	**59**	**59**	**59**	**59**	**59**	**50**	**9**	**59**	**9**	**50**	**59**	**59**	**59**	**59**	**59**	**59**	**9**	**50**	**59**	**59**	**59**	**59**
